# Dissection of the global responses of mandarin fish pyloric cecum to an acute ranavirus (MRV) infection reveals the formation of serositis and then ascites

**DOI:** 10.1128/jvi.02308-24

**Published:** 2025-05-14

**Authors:** Wenfeng Zhang, Yong Li, Xiaosi Wu, Qianqian Sun, Yuting Fu, Shaoping Weng, Jianguo He, Chuanfu Dong

**Affiliations:** 1 State Key Laboratory of Biocontrol/School of Life Sciences of Sun Yat-sen University 200651 , Guangzhou, China; 2 Southern Marine Science and Engineering Guangdong Laboratory (Zhuhai) 590852 , Zhuhai, China; 3 Institute of Aquatic Economic Animals and Guangdong Province Key Laboratory of Aquatic Economic Animals, Sun Yat-Sen University 26469 , Guangzhou, China; 4 Zhuhai Modern Agriculture Development Center, Zhuhai, China; Northwestern University Feinberg School of Medicine, Chicago, Illinois, USA

**Keywords:** mandarin fish ranavirus (MRV), ascites syndrome, pyloric cecum, serositis, ScRNA transcription

## Abstract

Mandarin fish ranavirus (MRV), a new member of the species *Ranavirus micropterus1*, sharing over 98% whole-genome nucleotide identity with the well-known largemouth bass virus (LMBV), is a distinct member of the genus *Ranavirus* within the family *Iridoviridae*. Our recent work showed that acute MRV infection predominantly affects the pyloric cecum, a critical visceral organ in mandarin fish, and was hypothesized to drive the characteristic external clinical sign of severe ascites. In this study, we reveal that acute MRV infection initially targets the serosal layer of the pyloric cecum of mandarin fish, leading to rapid progression into fibrinous serositis characterized by serosal hypertrophy, fibrosis, hyperemia, edema, and tissue adhesions. Using single-cell RNA sequencing, we dissect the cellular composition of epithelial, immune, and stromal populations, identifying significant enrichment of macrophages and granulocytes, alongside T and natural killer cells, as key mediators of acute cytokine and inflammatory responses. Then, robust experimental evidence demonstrates that MRV infects specific immune cell subsets of T and B cells and stromal cells of fibroblasts, myofibroblasts, endothelial cells, and pericytes, resulting in upregulation of genes and pathways associated with extracellular matrix (ECM) formation, collagen biosynthesis, and vascular remodeling in the hyperplastic serosal zone. Additionally, both host-derived type V collagens and MRV-encoded collagens are implicated in ECM formation in the hypertrophic serosa. Collectively, this study provides a comprehensive single-cell resolution analysis of the pyloric cecum’s response to acute MRV infection and highlights virus-driven serositis as the underlying cause of severe ascites in mandarin fish.

**IMPORTANCE:**

The pyloric cecum is a vital digestive and immune organ in many bony fish species, including the mandarin fish, a carnivorous species with an exceptionally developed pyloric cecum comprising 207–326 ceca per individual. While MRV/LMBV infects various fish species, severe ascites is uniquely observed in infected mandarin fish. This study demonstrates that acute MRV infection induces fibrinous serositis in the pyloric cecum, characterized by hyperemia, edema, and hyperplasia, ultimately resulting in ascites and mortality. Leveraging single-cell RNA sequencing, we provide a detailed landscape of the cell types affected or involved in the inflammatory response, revealing their roles in the pathogenesis of serositis. These findings advance our understanding of MRV-induced pathology and its species-specific manifestations.

## INTRODUCTION

Iridoviruses are large, icosahedral viruses possessing a single molecule of double-stranded DNA (dsDNA). Their genomes range from 99 kilobases (kb) to 220 kb, encoding 97 to 221 open reading frames (ORFs) (1, 2). According to the latest report of the International Committee on Taxonomy Viruses (https://ictv.global/taxonomy), the family *Iridoviridae* comprises seven genera. Among these, four genera (*Chloriridovirus, Iridovirus, Decapodiridovirus, and Daphniairidovirus*) infect invertebrates such as insects, crustaceans, as well as mollusks, while the remaining three genera (*Ranavirus, Lymphocystivirus*, and *Megalocytivirus*) infect a variety of cold-blooded vertebrates (3). Among vertebrate-infecting iridoviruses, ranavirus stands out for its broad host range, infecting bony fish, amphibians, and reptiles. Ranavirus infections often cause high morbidity and mortality in commercially valuable fish, amphibians, and threatened wildlife (4–8).

The clinical symptoms of ranavirus infections are generally systemic, characterized externally by multifocal cutaneous hemorrhages and erythema and internally by necrosis of hematopoietic tissues, vascular endothelium, and epithelial cells, along with hemorrhage and the formation of intracytoplasmic basophilic inclusion bodies (9–12). The affected organs often include the liver, spleen, and kidney (pronephros and mesonephros) and occasionally the gastrointestinal mucosa, lymphoid tissue, and neuroepithelial tissue (4, 6, 11–14). Mandarin fish ranavirus (MRV) and largemouth bass virus (LMBV) represent isolates from distinct species of fish and are considered strains/variants of the species *Ranavirus micropterus1* (genus *Ranavirus*, family *Iridoviridae*) (2). The clinical symptoms of LMBV infection are generally like those of other ranaviral infections. Under laboratory conditions, the featured external sign of LMBV-infected either largemouth bass (*Micropterus salmoides*) or smallmouth bass (*M. dolomieu*) was the development of ulcerative dermatitis and necrotizing myositis at the site of infection. In addition, LMBV has been reported by Hanson et al. to primarily target the swim bladder in largemouth bass, whereas Zilberg et al. demonstrated that it can also cause fibrinous peritonitis and serosal inflammation between the gastrointestinal tract and liver in juvenile fish (13, 15–17). By contrast, the acute MRV infections in mandarin fish (*Siniperca chuatsi*) are marked by severe ascites, a unique external clinical feature not observed in other *ranavirus* infections (18, 19).

A recent histopathological investigation showed that the pyloric cecum of mandarin fish was the visceral organ with the highest viral load upon acute MRV infection and then proposed that MRV might be a digestive tract pathogen in mandarin fish, which might contribute to the ascites syndrome (20). The pyloric cecum is located in the second segment of the gastrointestinal tract and serves critical roles in digestion, nutrient absorption, and immune responses in bony fish (21). While Aristotle first described the pyloric cecum as early as in 345 BCE (22, 23), its precise functions have been historically debated. Modern studies have clarified its roles in food storage, digestion, and as an active immune organ (23–25). Mandarin fish possess a highly developed pyloric cecum, with each individual hosting 207–326 ceca (26), a stark contrast to the 25 ceca observed in largemouth bass (23). Our recent study showed that the pyloric cecum of mandarin fish is the primary targeted internal organ upon acute MRV infection, which has not been documented in other ranaviral infections (20). By contrast, in LMBV-infected largemouth bass, the over-inflated swim bladder with thick, yellow, or brown exudates was described as one of the most featured internal clinical signs (27). Despite these observations, the mechanisms underlying MRV-induced ascites and the contrasting organotropism of MRV and LMBV remain poorly understood.

Single-cell RNA sequencing (scRNA-seq) has transformed our understanding of cellular heterogeneity in various fields, including immunology, oncology, and virology (28–31). This technique enables high-resolution analysis of transcriptional states at the single-cell level, offering unique insights into viral tropism, host cell responses, and virus-host interactions (32–38). In this study, we revisited the histopathological changes in the pyloric cecum of mandarin fish following acute MRV infection and applied scRNA-seq to elucidate the transcriptional landscapes of infected and uninfected pyloric cecum cells. Our findings identified targeted immune (B and T lymphocytes) and stromal cells (fibroblasts, myofibroblasts, endothelial cells, and pericytes) as key contributors to the inflammatory cytokine response and the development of severe fibrinous serositis. Together, these processes drive ascites syndrome and mortality in infected mandarin fish.

## RESULTS

### Pyloric ceca serve as the internal tissue with the highest viral load, and the serosa layer bears the primary infection

**Fig 1:**
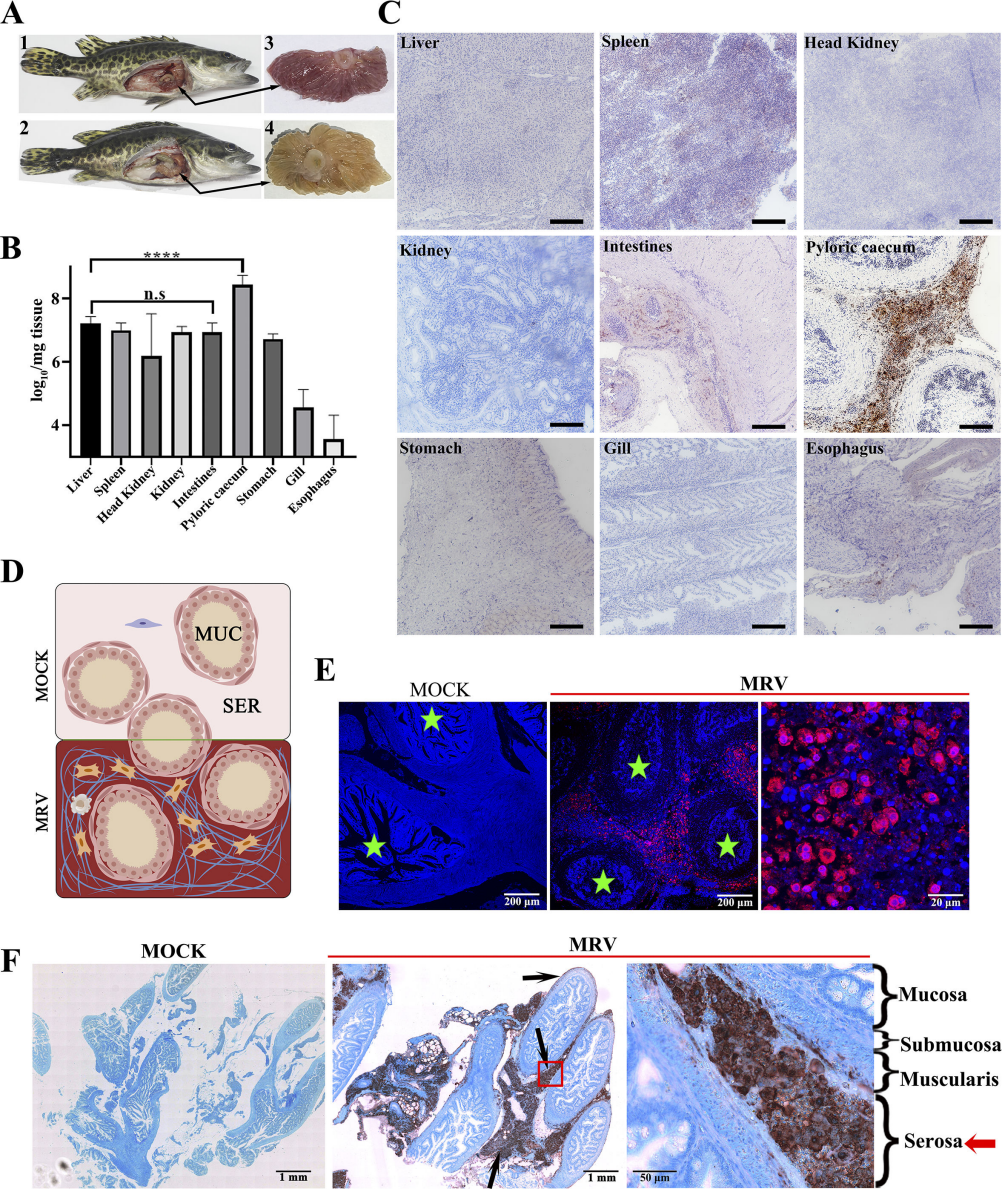
The primary infection site of MRV in the pyloric caeca of mandarin fish. (**A**) The autopsy of MRV-infected mandarin fish. (**A1**) and (**A3**), the diseased fish was characterized by severe hemorrhage in pyloric ceca. (**A2**) Healthy fish with normal pyloric ceca (**A4**). (**B**) Tissue distributions of MRV in various tissues by absolute RT-qPCR (*n* = 3). The highest viral load was determined in infected pyloric ceca. Statistical significance between pyloric ceca and liver (the second highest viral load) groups is denoted by ****, where the *P* value was < 0.0001 using one-way ANOVA and Tukey’s post hoc test. n,s, no significant. (**C**) Localization of MRV detected by immunohistochemistry (IHC). The strongest MRV-positive signals were found in infected pyloric ceca. Scale bars, 200 µm. (**D**) Schematic drawing of the cross-section of mandarin fish pyloric ceca. MUC, mucosa; SER, serosa. (**E**) Histo-immunofluorescence (HIF) of mandarin fish pyloric ceca infected with MRV. Numerous red fluorescence signals indicate MRV-infected cells by anti-MRV mAb 1C4. The stars indicate the digestive cavity. Scale bars of healthy fish and infected fish (left), 200 µm. Scale bars of infected fish (right), 20 µm. (**F**) Immuno-histochemistry (IHC) staining of MRV-infected pyloric ceca. Arrows indicate the sites stained by anti-MRV mAb 1C4. The serosa layer was observed as the site with the highest viral load in the pyloric cecum. Scale bars of healthy and infected fish (left), 1 mm. Scale bars of infected fish (right), 50 µm.

Through a series of experiments including necropsy observation, real-time quantitative polymerase chain reaction (RT-qPCR), as well as immunohistochemistry (IHC) and immunofluorescence (IF), we systematically examined the infection sites and replication patterns of the acute MRV infection (Fig. 1; Fig. S1). The results revealed that the MRV infection caused severe congestion and edema in the pyloric ceca, highlighting its high susceptibility to the virus (Fig. 1A). Both RT-qPCR and IHC analysis showed significantly higher viral loads and stronger MRV signals in the pyloric ceca compared to other tissues (Fig. 1B and C). Microscopically, the healthy pyloric cecum displays a layered structure comprising mucosal, submucosal, mucosal muscle, and the outer serosal layers (Fig. 1D and F). In contrast, the transverse sections of infected pyloric ceca, stained with anti-MRV monoclonal antibodies, showed a high concentration of MRV-positive signals in the serosal structures by IF (Fig. 1E). Few MRV-positive signals were detected in the mucosal, submucosal, or mucosal muscle layers, indicating a specific tropism of MRV to the serosa. Furthermore, in contrast to the uninfected healthy pyloric ceca, the infected pyloric ceca revealed significant expansion of the serosal structure, providing a favorable environment for extensive viral replication (Fig. 1F). Temporal analysis of the infection revealed a progressive formation of proliferative serosa with MRV infection (Fig. S1). At 1 day post-infection (dpi), slight hyperplasia was observed along the serosal layer. By 3 dpi, obvious hyperplasia filled the major gaps among pyloric ceca, and by 5 dpi, severe hyperplasia had completely filled these gaps. IHC and IF analyses confirmed that the serosal layer bears the primary MRV infection as early as 1 dpi, with high concentrations of MRV detected at 3 and 5 dpi, especially in the hypertrophic regions of infected pyloric ceca. In contrast, the interface of uninfected, healthy pyloric ceca remained clear and intact, with no hyperplasia or adhesion. All these results revealed that MRV infection in mandarin fish shows a unique tropism toward the pyloric ceca, among which, the serosal structure bears the primary infection of MRV.

### Overview of the cell types of pyloric ceca by scRNA-seq

**Fig 2:**
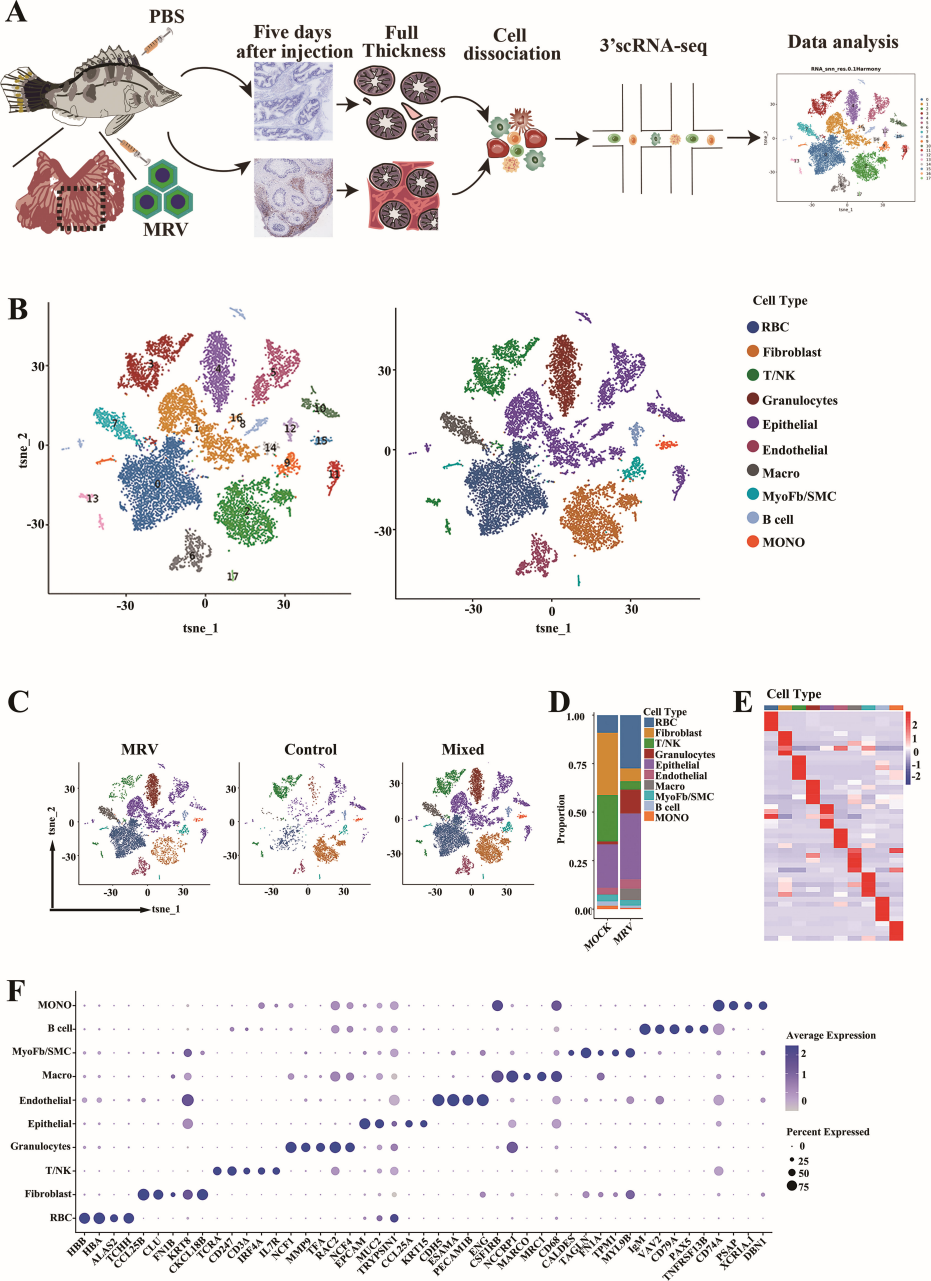
Cell sorting and categorization of cell types of mandarin fish pyloric ceca. (**A**) Overall strategy for cell sorting and single-cell sequencing data analysis. (**B**) The tSNE plot clustering of cells (left): different cell clusters are color-coded; The tSNE plot showed the expression of pan marker genes in distinct cell clusters (right), and the gene expression level is color-coded. (**C**) The tSNE plots align the clusters between control and MRV-infected mandarin fish. (**D**) Bar plots showing the proportion of each cluster in control and MRV-infected groups. (**E** and **F**) Average expression level and prevalence of selected major markers used to annotate the major cell types. Heatmap (**E**) and dot plot (**F**) of the expression of the marker genes in each cell type.

To comprehensively understand the cellular heterogeneity of pyloric ceca, scRNA-seq was performed on two uninfected (control) and two MRV-infected mandarin fish (accession number: PRJNA1225909) (Fig. 2A). To ensure robust data analysis, cells with fewer than 400 detected genes or more than 25% mitochondrial unique molecular identifier (UMI) counts were excluded. After eliminating data outliers based on the number of reads per cell (nUMI), the number of genes detected per cell (nGene), and thresholds for mitochondrial RNA genes, a total of 4,807 and 10,777 pyloric cecum cells were obtained from the control (C) and MRV-infected mandarin fish, respectively (Fig. S2). To explore the cellular landscape, we applied T-distributed stochastic neighbor embedding (tSNE) reduction and unsupervised cell clustering, leading to the identification of eighteen cell clusters (Fig. 2B). Classification based on unique transcription profiles and the top five expressed genes in each group allowed us to categorize these clusters into epithelial cells, immune cells, stromal cells, and red blood cells. Utilizing well-established marker genes (Fig. 2E and F; Fig. S3), we classified two major epithelial cell types (EPCAM) as ciliated epithelial cells (expressing CXCR4B and CCL25a) and secreting cells (expressing CHIA1, PYYA, and TRYPSIN). Within the immune cell population, five distinct cell types were specified: T/NK cells (expressing TCRα, CD247, and CD3δ), granulocytes (expressing MMP9 and NCF1), macrophages (expressing CSF1RB, MRC1, and MARCO), B cells (expressing IgM, Pax5, and CD79), and monocytes (expressing GRNA and MCP-1). Furthermore, three stromal cell types were also identified: fibroblasts (expressing FGFR2 and FN1B), endothelial cells (expressing CDH5 and CLDN7), and myofibroblast and smooth muscle cells (expressing ACTA2 and MYH11). Notably, red blood cells (expressing HBA and HBB) were abundantly present in the pyloric cecum of MRV-infected mandarin fish (Fig. 2B). With infection of MRV, the number and proportion of each cell subpopulation changed, as shown in Fig. 2C and D.

### Identification and characterization of epithelial subtypes and stromal subtypes

**Fig 3:**
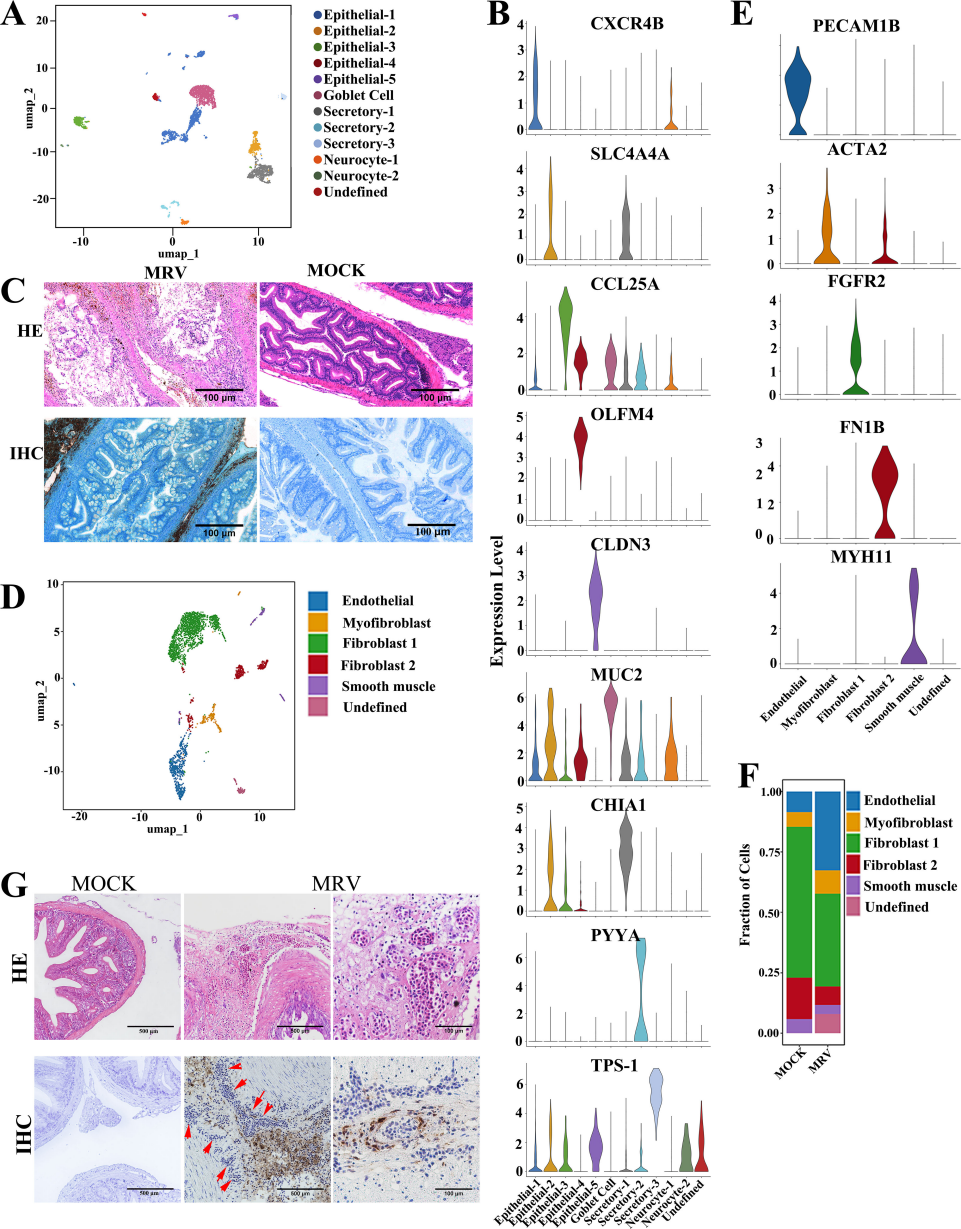
Subtypes of epithelial cells and stromal cells in the pyloric ceca. (**A**) Re-clustering 4,500 epithelial cells of pyloric cecum identified 12 subclusters, shown in the UMAP space. (**B**) Average expression levels and prevalence of major markers used to identify non-secretory epithelial cells and secretory cells of the 12 epithelial cell subtypes. (**C**) H&E staining and IHC staining of mucosa using anti-MRV mAb; The lower left image in Fig. 3C is a magnified region from the whole-slide IHC image of Fig. 1F (the center image), highlighting the absence of MRV signals in the digestive tract. (**D**) The UMAP plot shows the five stromal cell subtypes identified in pyloric ceca. Different stromal cell subtypes are color-coded. (**E**) Violin plots showing the average expression level and prevalence of selected major markers used to annotate the stromal cell types. (**F**) Bar plots showing the proportion of each cluster in the stromal cells from control and MRV-infected groups. (**G**) H&E and IHC of mandarin fish pyloric ceca infection with MRV. The arrows indicate the hypertrophic serosa filled with microvessels. Scale bars, 100 µm.

By re-clustering approximately 4,500 epithelial cells identified in the global analysis, twelve distinct molecularly defined subtypes were identified (Fig. 3A). The gene expression of tSNE plots was generated to identify marker genes for each epithelial subpopulation. Among these, non-secretory epithelial cells were designated Epithelial-1 to Epithelial-5, secretory cells were designated as Secretory-1 to Secretory-3, and goblet cells based on isotype-specific RNA markers. Additionally, two enteric nervous system cell subtypes were designated: Neurocyte-1 and Neurocyte-2 (Fig. 3B; Fig. S4A). Given that few MRV-positive signals were observed in the mucosa of the pyloric cecum cavity (Fig. 1 and 2), epithelial cells in the pyloric ceca were excluded as MRV target cells. Although MRV did not infect the epithelial layer of the pyloric cecum and the villi remained structurally intact, partial adhesion, epithelial exfoliation, and infiltration of inflammatory cells into the mucosal muscle were observed. These findings suggest that MRV infection may induce mucosal damage in the pyloric ceca, affecting both epithelial cell injury and subsequent recovery via cell proliferation (Fig. 3C).

The global clustering involved 3,410 stromal cells (Fig. 2B), which were re-clustered and categorized into six distinct stromal cell types (Fig. 3D and E). These cell types include endothelial, myofibroblasts, fibroblast-1, fibroblast-2, smooth muscles, and an undefined cell. Among them, there were significant increases in endothelial cells, myofibroblasts, and the undefined stromal cells after MRV infection. In contrast, other cells showed varying degrees of reduction (Fig. 3F). The t-SNE plot also showed the expression of selected marker genes which were enriched in subsets (Fig. S4B). Through pathological observation and virus tracing in MRV-infected mandarin fish, we once again observed the predominant localization of the virus in the serosal layer, leading to upregulation of expression of numerous genes associated with extracellular matrix (ECM) deposition and remodeling, resulting in severe congestion, edema, and hyperplasia (Fig. 3G; Fig. S7A).

### Compositions and functions of immune cell subpopulations

**Fig 4:**
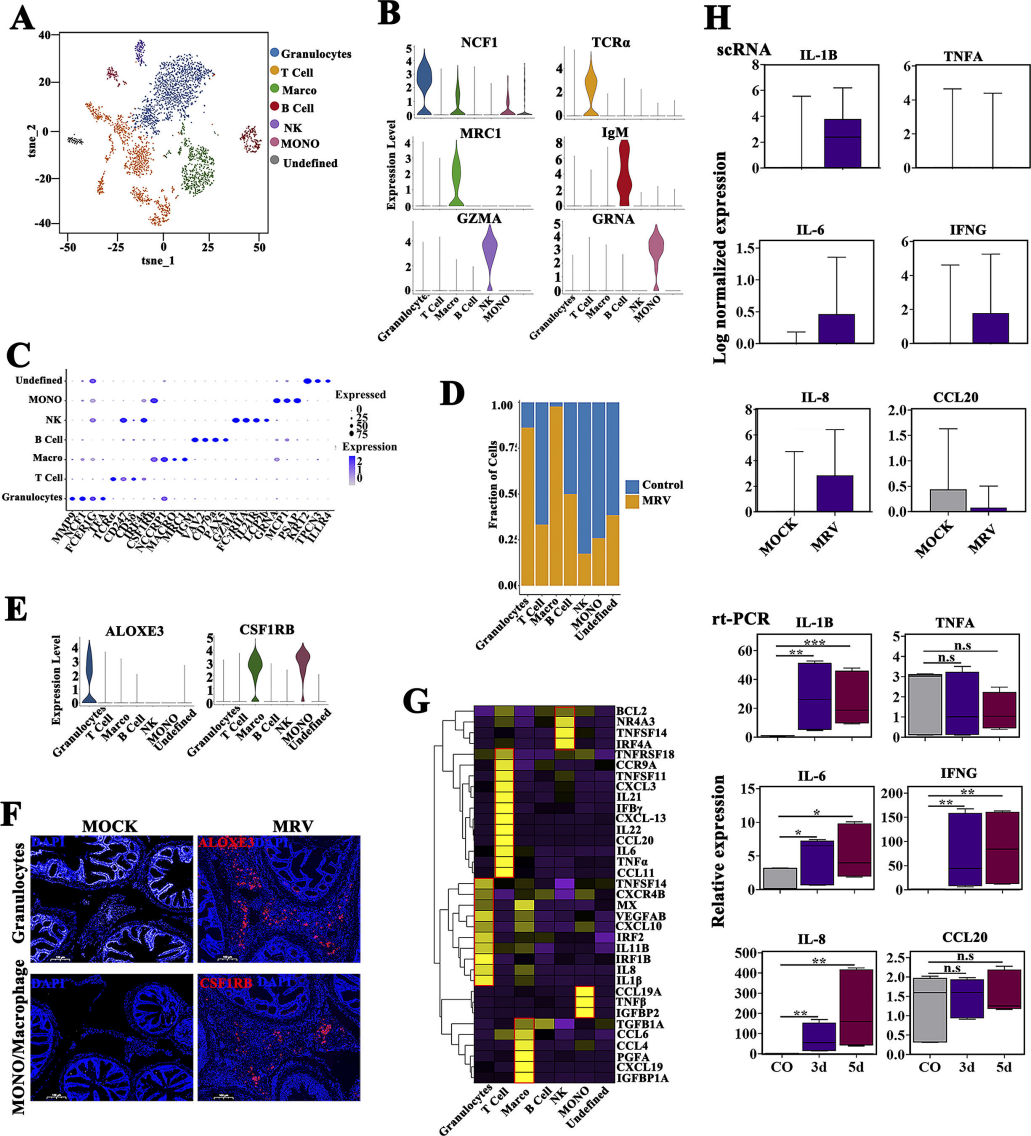
Immunological features of immune cell subsets upon MRV infection. (**A**) The tSNE plot shows the seven immune cell subtypes identified in pyloric ceca. Different immune cell subtypes are color-coded. (**B** and **C**) average expression level and prevalence of selected major markers used to annotate the immune cell types. Violin map (**B**) and dot plot (**C**) of the expressions of the marker genes in each immune cell type. (**D**) Bar plots showing the proportion of each immune cell cluster in control and MRV-infected groups. (**E**) Violin plot showing the expression of granulocytes (ALOXE3) and macrophage/monocyte (CSF1RB) marker across the seven immune cell clusters. (**F**) FISH images showing the expression of granulocytes subtype marker ALOXE3 (Up) and macrophage/monocytes subtype marker CSF1RB (Down), Scale bar, 100 µm. (**G**) Heatmap of cytokine expressions among seven immune cell subtypes. (**H**) Boxplots of cytokine expressions based on scRNA-seq and RT-qPCR profiling for healthy control and MRV-infected mandarin fish. Data are expressed as means ± SD (**P < 0.01 or ***P < 0.001).

Upon re-clustering approximately 4,000 immune cells from both infected and healthy mandarin fish, six distinct immune cell subtypes were identified (Fig. 4A). Gene expression bubble maps were generated to highlight marker genes for each immune cell subpopulation, and granulocytes, T cells, macrophages, B cells, NK cells, and monocytes were obtained (Fig. 4B and C; Fig. S5A). Among these cell types, T cells and B cells emerge as the predominant immune cell populations in pyloric ceca of healthy mandarin fish (Fig. 4D). In response to MRV infection, the numbers of macrophages and granulocytes significantly increased, while T cell numbers significantly decreased. The count of B cells remained largely unchanged (Fig. 4D). Fluorescence *in situ* hybridization (FISH) results also confirmed that numerous ALOXE3^+^ granulocyte and CSF1RB^+^ macrophage/monocyte signals were observed in the hypertrophic serosal region (Fig. 4E and F). By contrast, few ALOXE3^+^ and CSF1RB^+^ signals were found in the healthy pyloric cecum.

To further investigate alterations in gene transcription in immune cells following MRV infection, we compared gene expression patterns between control and MRV-infected cells. Upon viral infection, the expression of most immune-related genes was significantly upregulated. These differentially expressed genes (DEGs) are involved in leukocyte activation and are associated with chemokine signaling pathways (Fig. S5C). Subsequently, we examined the inflammatory characteristics of each hyperinflammatory cell subtype to identify potential sources of cytokine production. Distinct pro-inflammatory cytokine gene expressions were identified in each cell subtype (Fig. 4G), suggesting that diverse cell types secrete distinct pro-inflammatory factors, contributing to cellular storm phenomena through multiple mechanisms involving cytokine storm induction. We collected scRNA-seq data along with qPCR test results (Table S1), both of which confirmed elevated levels of several pro-inflammatory cytokines, such as IL-1β, IL-8, IL-6, and IFNG, in the MRV-infected group. However, not all pro-inflammatory factors exhibited increased expression, including TNFα and CCL20 (Fig. 4H).

### Spatial location of infected pyloric ceca single cells

**Fig 5:**
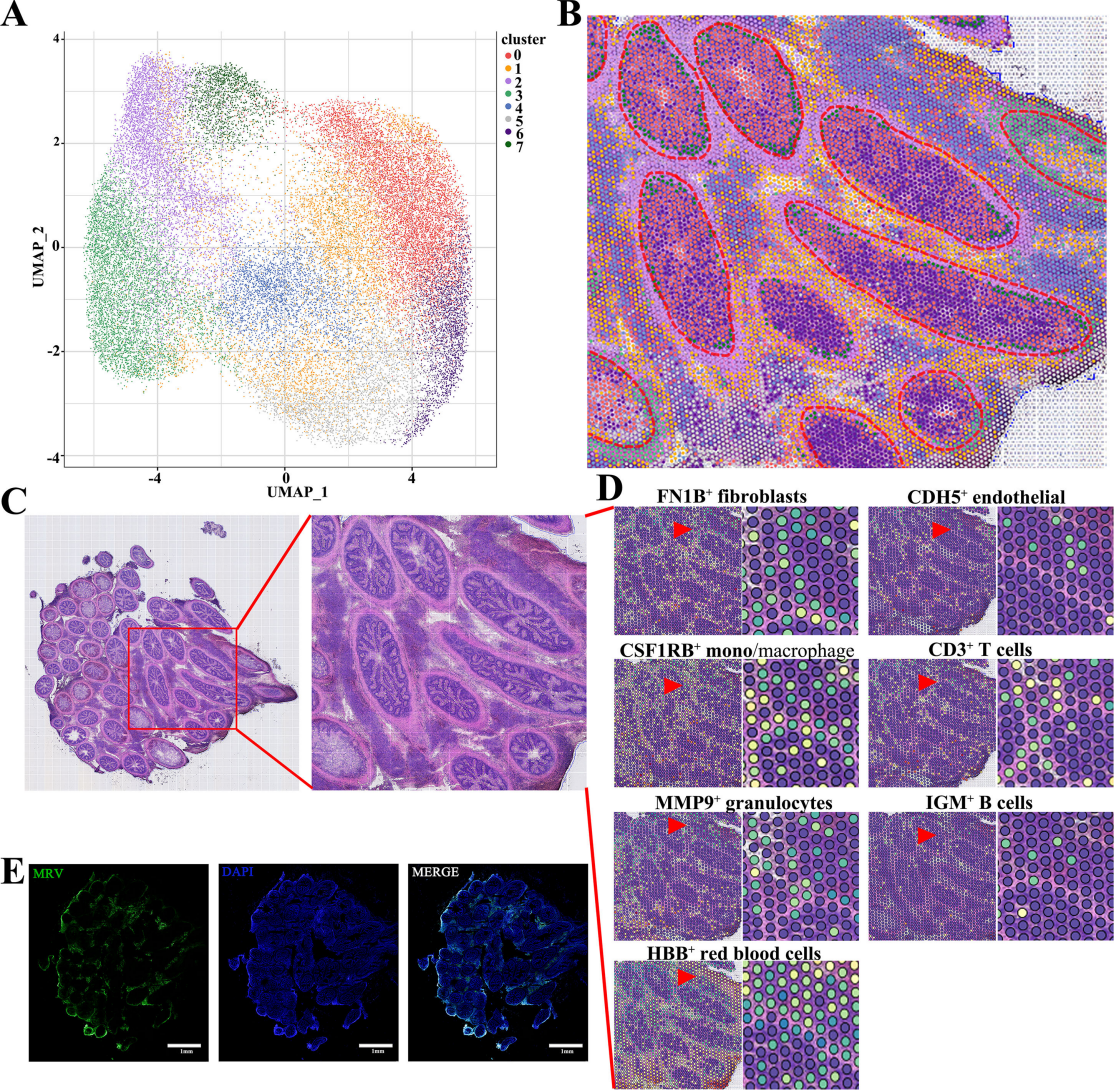
Spatial location of infected pyloric ceca single cells. (**A**) UMAP plot displaying dimensionality reduction clustering analysis of MRV-infected pyloric ceca by spatial transcriptomics. (**B**) Spatial transcriptomics data showing the distribution of cell clusters in MRV-infected pyloric ceca in the pathological section (partial). The red circle outlines the individual pyloric ceca. (**C**) Histopathologic microscan of the section of MRV-infected pyloric caeca. The H&E image and the ST are derived from the same tissue section. The red box indicates the magnified area. (**D**) Expression of selected cell markers identified by scRNA-seq in hyperplastic serosa. The representative views indicated by the triangular arrows are shown in the enlarged image on the right. (**E**) IF staining of MRV (green) in an adjacent section, scale bar, 1 mm.

Due to viral infection causing cell lysis and significant extracellular matrix accumulation (especially in the serosal layer), single-cell RNA sequencing does not fully represent the actual cell population proportions. Additionally, single-cell RNA-seq lacks a spatial context, making it difficult to identify the cell types where viral replication occurs. Spatial transcriptomics allows us to identify the proliferating cell types in the hyperplastic serosa and determine their origins. To further investigate the cellular composition of hyperplastic serosa, spatial transcriptomics (ST) was employed to map the spatial distribution of scRNA-seq data from MRV-infected pyloric ceca. Analysis of transcriptional signatures of ST spots identified seven spot clusters in the slide, which were observed to be mapped to discrete anatomical regions (Fig. 5A and B). Using the scRNA-seq atlas as a reference, we performed factor analysis to determine the likely single-cell composition of each spot, thus spatially localizing all scRNA-seq clusters. This approach revealed significant differences in cell composition between the gastrointestinal tract, muscle layer, and hyperplastic serosa (Fig. 5B and C; Fig. S6A). Furthermore, we observed that the hyperplastic serosa predominantly consisted of fibroblasts, endothelial cells, monocyte/macrophages, T cells, granulocytes, B cells, and erythrocytes (Fig. 5D; Fig. S6B). Global IF scanning further confirmed that MRV was primarily localized within the hyperplastic serosal region (Fig. 5E). These findings underscore the heterogeneity of the hyperplastic serosa and provide insights into why MRV specifically targets this area, rather than the gastrointestinal tract.

### MRV mainly infects B cells, T cells, myofibroblasts, fibroblasts, endothelial cells, and pericytes

**Fig 6:**
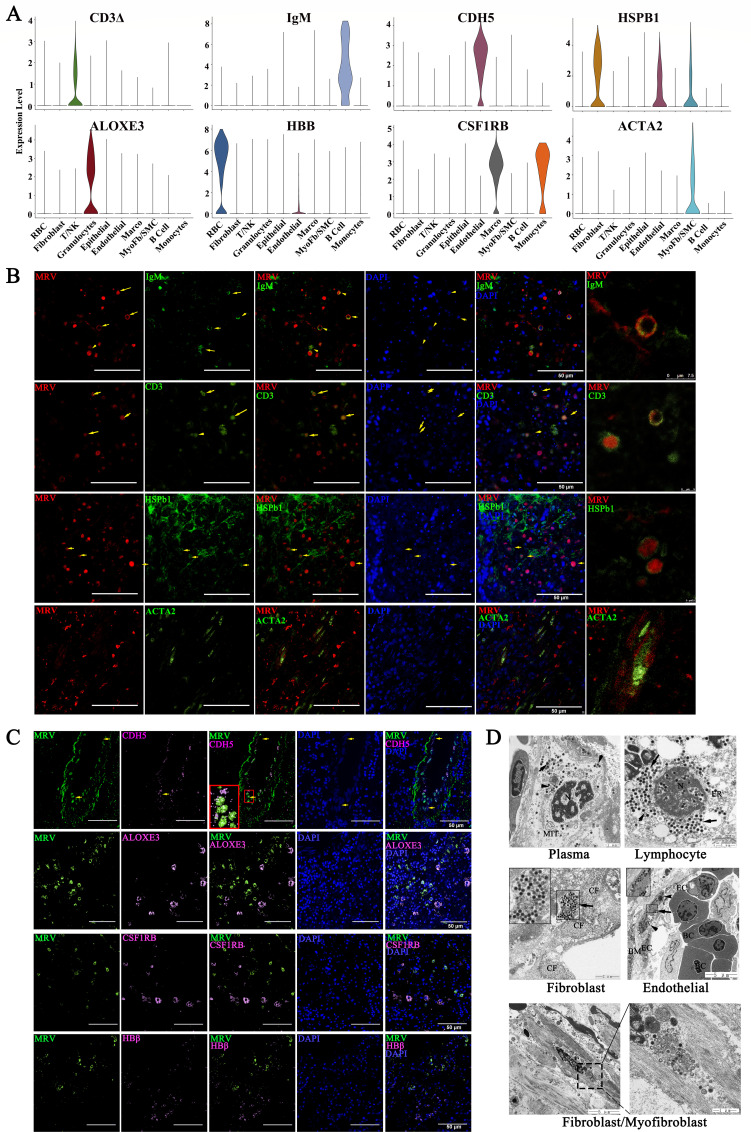
Colocalization of MRV signals and specific markers. Dual-color IF and FISH staining to validate the selected marker and MRV signal. (**A**) Expression levels of selected markers used to identify the selected cell subtypes. (**B**) Dual-color IF staining to validate colocalization of the the selected markers IgM, CD3, HSPb1, ACTA2 (green) and MRV (red). The nucleus was stained with DAPI (blue), Scale bar, 50 µm. (**C**) Dual-color FISH and IF staining to validate the colocalization of the selected markers CDH5, ALOXE3, CSF1RB, HBβ (pink), and MRV (green). Selected markers were identified by the corresponding probe and signal probe (CY5). MRV was identified by anti-MRV monoclonal antibody and secondary goat anti-mouse IgG antibody coupled with AlexaFluor 488 (green). The nucleus was stained by DAPI (blue), Scale bar, 50 µm. The arrows indicate the colocalization of cells. (**D**) Transmission electron micrograph of five kinds of target cells, BC: blood cell, EC: endothelial cell, BM: basement membrane, CF: collagenous fiber, N: nucleus, Mit: mitochondria, ER: endoplasmic reticulum. The arrows indicated the virions. The scale is shown in the figure.

**Fig 7:**
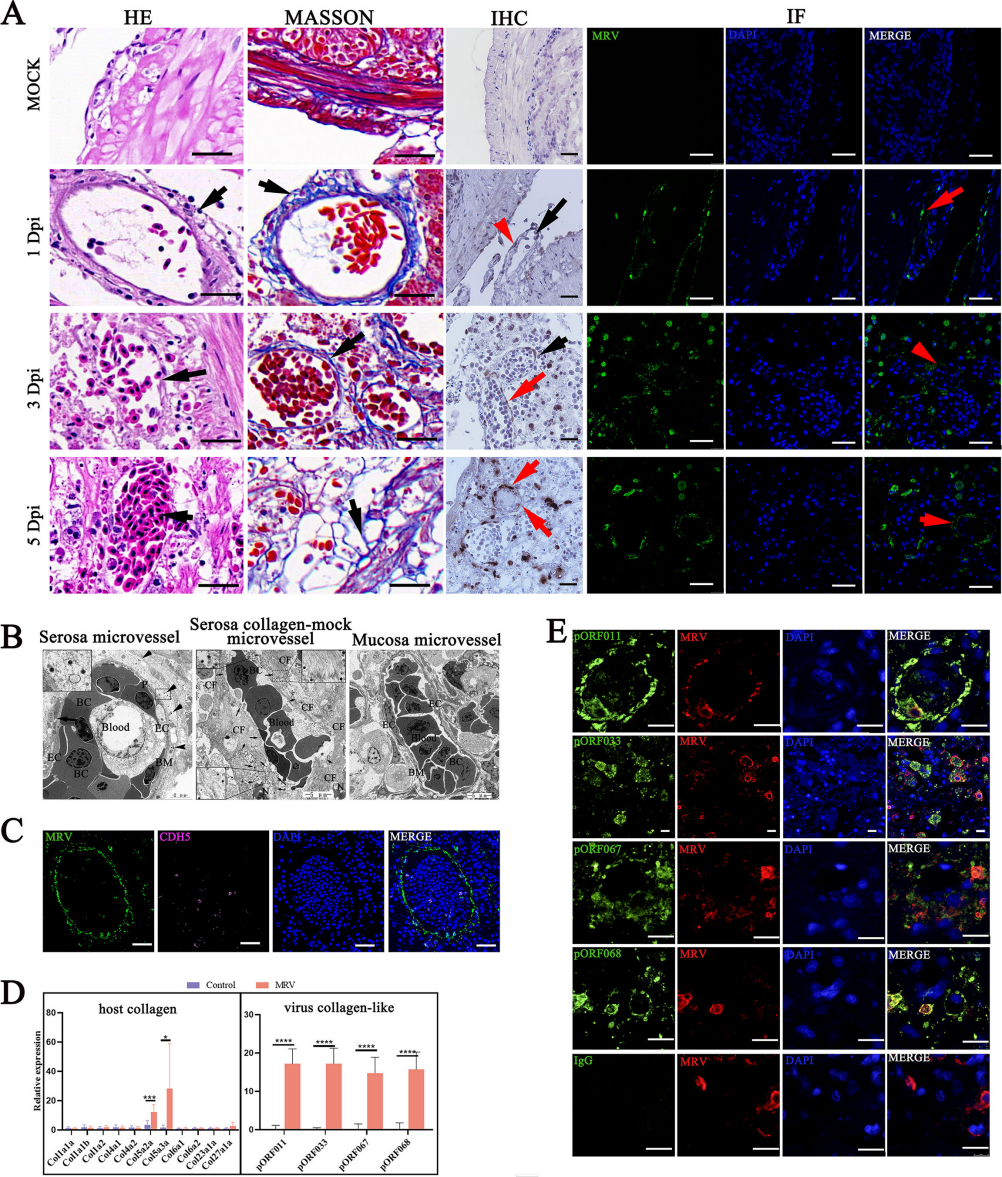
Serosal fibrosis after MRV infection. (**A**) HE, IHC, IF, and MASSON staining observation of pyloric ceca serosa of MRV-infected mandarin fish at 1, 3, and 5 dpi, scale bar, 10 µm; black arrows indicate the microvessels; red arrows indicate the MRV virion signals. Scale bar, 20 µm. (**B**) Transmission electron micrograph of MRV-infected pyloric ceca; (B left) depicts a comprehensive cross-section of microvessels, exhibiting virion-infected endothelial cells and pericytes; scale bar, 2 µm; (B middle) shows a collagen-based vascular lumen devoid of endothelial cells, filled with blood cells, and scattered surrounded by virions; scale bar, 5 µm; (B right) showcases intact and distinct mucosal microvessels due to the absence of viral infection in the mucosal layer; scale bar, 5 µm. BC: blood cell, EC: endothelial cell, BM: basement membrane, CF: collagenous fiber, N: nucleus. Arrowheads indicated the virions. (**C**) FISH data showing the localization of virions (green) and CDH5-positive endothelial cells within the microvascular-like lumen. The arrows indicate the shed endothelial cells. Scale bar, 20 µm. (**D**) The relative mRNA levels of eleven host-encoded collagen (left) and four MRV-encoded collagen-like proteins (right) by RT-qPCR. (**E**) Colocalization of MRV (red) and collagen-like pORF011, pORF033, pORF067, and pORF068 (green) in infected pyloric cecum by IF. The nucleus was stained with DAPI (blue); scale bar, 10 µm. Data are expressed as means ± SD (**P < 0.01 or ***P < 0.001).

The target cells of MRV infection in the pyloric cecum were systematically analyzed using scRNA-seq. Comparison of scRNA-seq data before and after MRV infection revealed a significant increase in the proportion of red blood cells (HBB), epithelial cells (EPCAM), granulocytes (ALOXE3), endothelial cells (CDH5), myofibroblasts (ACTA2), and macrophages (CSF1RB) relative to uninfected cells. Conversely, the proportion of fibroblasts (HSPB1) and T cells (CD3delta) decreased significantly compared to uninfected controls, while the ratio of B cells (IgM) and monocytes remained relatively unchanged (Fig. 2D and 6A). Epithelial and muscle cells were excluded from further analysis due to the absence of viral signals in these layers, as observed by IHC and IF (Fig. 1; Fig. S1). Dual-color IF analysis revealed the colocalization of MRV signals with HSPB1, IgM, CD3, and ACTA2, indicating that MRV primarily targets fibroblasts, myofibroblasts, T cells, and B cells (Fig. 6B). Further studies using FISH and IF identified the colocalization of endothelial cells (CDH5), red blood cells (HBB), and macrophages (CSF1RB) with MRV. The results revealed that the CDH5^+^ signal was colocalized with the MRV signal predominantly within blood vessels (Fig. 6C). Vascular structure analysis also confirmed that MRV also infects pericytes (Fig. 6D; Fig. 7B). No colocalization between ALOXE3^+^, CSF1RB,^+^ or HBB^+^ signals and MRV signals was observed (Fig. 6C), suggesting that none of granulocytes, macrophages/monocytes, or red blood cells were MRV-targeted cells. TEM further supported these findings, revealing the presence of numerous virions in plasma-like cells, lymphocyte-like cells, fibroblasts/myofibroblasts, vascular endothelial-like cells, and pericyte-like cells (Fig. 6D and 7B). In summary, MRV predominantly targets myofibroblasts, fibroblasts, endothelial cells, and pericytes, as well as B and T cells of the infected pyloric cecum.

### Acute MRV infection causes fibrosis of hypertrophic serosa

We identified the target cells of MRV infection through scRNA-seq. Following this, we sought to investigate the underlying mechanisms driving serosal hyperplasia in response to MRV infection. By further analyzing the pathological changes and Masson (collagen fibers appear blue and muscle fibers appear red) staining of pyloric ceca upon MRV infection, we found that the serosal hyperplasia of pyloric ceca was filled with the vessel-like luminal structures primarily composed of collagen fibers (Fig. 7A). With the progress in the infection, the peritoneal structure expanded outward, leading to an increase in the production of collagen fibers and the recruitment of matrix cells and immune cells to combat the viral infection. At the late stage of infection, the interwoven collagen fibers formed a type of extracellular matrix, providing a site for the recruited target cells to adhere (Fig. 7A). Differentially expressed genes associated with extracellular matrix formation, collagen-containing matrices, adherens junctions, and blood vessel morphogenesis were significantly enriched in myofibroblasts, fibroblasts, and endothelial cells in the MRV-infected group (Fig. S7A). TEM further revealed two distinct types of microvessels in the hypertrophic serosa: one consisting of normal endothelial cells and pericytes, which were infected by the virus (Fig. 7B**, left**), and the other displaying a vascular lumen composed entirely of collagen fibers. This second type showed a loose lumen structure, devoid of endothelial cells and basal membrane, but accompanied with significant accumulation of virions (Fig. 7B **middle**). The latter type was more prevalent in the later stages of infection. In contrast, the uninfected epithelial microvessels within the same sample maintained clear structural integrity (Fig. 7B **right**). FISH confirmed numerous MRV signals around these collagenous microvessels, but only a few endothelial cells were detected, suggesting that these structures are not true microvessels, but collagenous mock vascular structures (Fig. 7C).

To investigate the origin of the collagen fibers, we quantified the expression of 11 host collagen genes and observed a significant increase in the expressions of Col5a2a and Col5a3a following MRV infection, indicating that the produced collagen fibers were of type V (Fig. 7D). MRV whole-genome (accession number: OQ267588) analysis identified four viral proteins with collagen-like structures, namely, pORF011, pORF033, pORF067, and pORF068 (Fig. S7B). These viral proteins were highly expressed in the MRV-infected pyloric ceca (Fig. 7D). IF analysis showed strong expressions of pORF011, pORF067, and pORF068 around collagenous mock microvessels, suggesting their direct involvement in the formation of collagenous structures. Meanwhile, pORF033 was primarily localized in the cytoplasm, indicating potential involvement in other biological processes (Fig. 7E). Virion proteome of purified MRV confirmed that these four viral collagen-like proteins are nonstructural viral proteins. Taken together, these results suggest the possibility that MRV may actively encode collagen-like proteins to participate in the formation of the host extracellular matrix, potentially providing a niche for its replication. In the late stages of infection, the virus is released from infected cells, and due to the formation of loose fibrous serosal structures, interstitial fluid may leak into the abdominal cavity, possibly leading to ascites formation (Fig. 1A; Fig. S7C).

## DISCUSSION

The symptoms of acute MRV infection are systemic. Still, the primary cause of acute mortality in infected mandarin fish is infection-induced hyperemia and edema of pyloric ceca, along with the formation of severe ascites (19, 20). Investigating the specific site of MRV infection within the pyloric cecum and identifying the targeted cell types are critical for a deeper understanding of the virus’s pathogenesis. To address this issue, we revisited the histopathological changes in the pyloric cecum. Our findings again confirm that the pyloric ceca are the most affected visceral organs during acute MRV infection. Furthermore, the serosal structure of the pyloric cecum of mandarin fish was found to be the primary and possibly the sole site of MRV infection. As the disease progresses, the infected serosa rapidly expands outward, forming a hyperplastic zone and filling the space between the lobes of the pyloric ceca. The hyperplastic serosal zone becomes the most densely infected area, leading to hyperemia and edema of the pyloric ceca. We designate this outcome as acute serositis. In LMBV-infected largemouth bass, fibrinous peritonitis, characterized by fibrinous exudates containing numerous leukocytes and abundant cellular debris throughout the peritoneal cavity, is a prominent histopathological feature (13). Since the peritoneum and serosa are structurally similar, peritonitis or serositis can be considered characteristic histopathological outcomes in both LMBV-infected largemouth bass and MRV-infected mandarin fish. Notably, our recent study proposed that MRV might be a digestive tract virus, linked to ascites syndrome (19). However, this study clarifies that MRV infects only the serosal structure but not the pyloric cecum cavity itself, indicating that MRV is not a digestive tract virus. This conclusion aligns with the findings in LMBV-infected largemouth bass (13). In LMBV-infected largemouth bass, exudates are present on the ventral surface of the swim bladder contacting the peritoneal cavity. In contrast, MRV-infected mandarin fish develop severe ascites that fills the abdominal cavity. The mandarin fish’s more developed pyloric ceca (23), compared to the largemouth bass (26), may explain the distinct clinical and histopathological features observed in MRV infection. Overall, irrespective of multi-organ targeted peritonitis or pyloric cecum targeted serositis, the formation mechanism and characteristics of this inflammation response have never been studied. Thus, a comprehensive study based on scRNA-seq was conducted to address this issue.

For scRNA-seq analysis, we identified 10 distinct cell subtypes in the pyloric ceca of mandarin fish, including epithelial cells, five immune cell types, three stromal cell types, and red blood cells. The scRNA-seq profiling revealed a marked enrichment of granulocytes and macrophages in MRV-infected fish. Macrophages, T cells, granulocytes, and NK cells play pivotal roles in the inflammatory response and exhibit a robust inflammatory reaction. Additionally, fibroblasts, myofibroblasts, T cells, B cells, endothelial cells, and pericytes are the primary MRV-targeted cells. Furthermore, MRV infection induces severe fibrotic hyperplasia in the serosa, with four collagen-like proteins contributing to the formation of fibrous structures.

Previous studies have extensively characterized the cell types in the gastrointestinal tract of humans and mice (39–41), as well as in the zebrafish intestine (42). Pyloric ceca, a distinctive and significant structure in fish digestive systems, plays an important role in vertebrate evolution (21, 22). However, knowledge of the cell types in fish pyloric ceca remains limited. In this study, we focused on MRV-infected mandarin fish juveniles (average weight 50 ± 5 g). Single cells from pyloric ceca were collected from two healthy and two MRV-infected fish, yielding 10,777 and 4,807, respectively. Considering the high mitochondrial content in gastrointestinal cells, the threshold for filtering mitochondria was set at 25%. As a result, each control sample contained approximately 2,400 cells, while each infected sample contained over 5,000 cells due to hypertrophic serosa. Additionally, cell type-specific markers were identified to enable precise classification of cell types. Major cellular classes, including epithelial cells, fibroblasts, muscle cells, endothelial cells, and immune cells, could be distinguished based on the expression of category-specific genes associated with their respective functions. In fish, cells from the pyloric ceca can be divided into epithelial cells and other types of cells based on the expression of the pan-epithelial cell marker EPCAM. Epithelial cells were further classified into secretory cells and non-secretory cells based on the secretion of specific proteins. Secretory cell markers are well-defined and functionally distinct. For instance, Secretory-1 expresses chitinase and aminopeptidase, which aid in digesting crustacean food, while EC2 expresses YY peptide. In addition to MUC2-secreting goblet cells, EC3 expresses trypsin 1–3 and elastase, key digestive enzymes. Non-secretory epithelial cells lack specific subtype markers, so we categorized them as epithelial-1 to epithelial-5 based on genes that are highly expressed in each subtype. The functions of these subgroups were preliminarily speculated, though further data are needed to refine their classification. Overall, MRV infection induces damage to these epithelial cells; however, these cells are not permissive to MRV infection.

Beyond its digestive function, the gastrointestinal tract plays a crucial role in immunity, with the pyloric ceca of bony fish serving as a key site for immune activity (43). Immune cells identified in the digestive tract of bony fish include T lymphocytes, B lymphocytes, dendritic cells, macrophages, and granulocytes, all contributing to intestinal immunity (44). Our scRNA-seq results revealed a rich presence of T lymphocytes (TCRα^+^), B lymphocytes (IgM^+^), granulocytes (NCF1^+^), macrophages (MRC1^+^), NK cells (GZMA^+^), and monocytes (GRNA^+^) in the pyloric ceca of healthy mandarin fish. Among these cell types, T cells, B cells, and NK cells comprised the majority of the immune cell population. Previous studies have shown that peritoneal recruitment and accumulation of NK cells occur three days after ranavirus infection, while lymphocyte recruitment is delayed until 6 dpi (44). In contrast, our study demonstrated that macrophages and granulocytes exhibited rapid responses to MRV infection, recruiting to the infected site within 3 dpi, while other immune cell types did not show significant enrichment. Nonspecific cytotoxic cells (NCC), a fifth type of fish-specific cytotoxic cells and evolutionary precursors of NK cells, were not detected in our study. The marker NCCRP1 was specifically expressed in macrophages and granulocytes, whereas granzyme showed specific expression in NK cells. NCC may have either disappeared in mandarin fish or may never have been present, suggesting that the immune system in this species may possess unique evolutionary traits among teleosts.

In contrast to higher vertebrates, fish are free-living organisms from the early embryonic stages of life, relying on their innate immune systems for survival. Nonspecific immunity is the important defense mechanism in fish, although their immune memory response is typically less developed than in higher vertebrates (45, 46). Ranavirus infection is often associated with a prominent host inflammatory response. Similar to viral infections in mammals, ranavirus-induced inflammation is a double-edged sword: while it is essential for viral clearance, it can also exacerbate disease progression and negatively impact host survival (47–49). Previous studies have observed that ranavirus infection induces the expression of inflammatory genes such as interleukin-1-β (IL-1β), tumor necrosis factor-α (TNFα), and the anti-inflammatory arginase-1 (Arg-1) (44, 50, 51). Following infection, interferon and the downstream protein Mx are rapidly expressed, though they fail to effectively counter the virus (52, 53). In this study, we found that the acute MRV infection rapidly initiated the increase in many inflammatory factors. Nearly all immune cell types, including granulocytes, macrophages, T cells, and NK cells, are involved in the secretion of these inflammatory factors. For example, granulocytes produce IL-1β and IL-8, while T lymphocytes secrete IL-6 and IFNγ, all of which increase significantly within 3 dpi. However, TNFα, secreted by T lymphocytes, does not exhibit significant changes post-MRV infection. These findings suggest that acute MRV infection triggers a strong inflammatory response in the pyloric ceca, aiming to mitigate tissue damage caused by the virus. However, these inflammatory responses are insufficient to clear the virus and may, in fact, worsen the disease and negatively affect host survival. In the case of the pyloric ceca, MRV infection of the serosa leads to severe serositis, characterized by the accumulation of numerous inflammatory cells and the secretion of inflammatory mediators. Additionally, many stromal cells, such as fibroblasts, myofibroblasts, endothelial cells, and pericytes, are recruited to form a hypertrophic serosal zone. Notably, B cells and T cells in the immune cell subpopulation, along with fibroblasts, myofibroblasts, endothelial cells, and pericytes in the stromal cell population, are the primary targets of MRV infection. As a result, MRV infection exacerbates hyperemia and edema within the hypertrophic serosal zone, induces severe ascites, and ultimately leads to high mortality.

ST provided deeper insights into the tissue-specific effects of MRV infection, particularly in the serosal layer. A key finding was the significant heterogeneity of proliferating cells in the hyperplastic serosa, which did not originate from the mucosal layer. This suggests that cell proliferation in the serosa is not due to local expansion of existing cells but may involve the recruitment of cells from other regions via the circulatory system. This helps explain why MRV specifically targets the serosal layer rather than the gastrointestinal tract. Additionally, we identified fibroblasts, endothelial cells, mononuclear macrophages, granulocytes, and others as the main cell types in the serosa, providing a basis for further studies on viral tropism. Notably, these cells are involved in immune responses and tissue repair, suggesting that MRV may exploit the host’s inflammatory and repair mechanisms to promote replication and spread. This insight offers a new perspective on viral pathogenesis, indicating that MRV may modulate the host’s immune response to its advantage. Furthermore, the ST data complement our single-cell RNA-seq findings, offering a more comprehensive view of the cellular landscape during MRV infection.

The epidemiology, pathogenesis, host antiviral response, and immune escape mechanisms of ranavirus have been extensively studied. However, there have been limited investigations into the specific target cell types of ranavirus infection (54–56). In chronic infections, very low copy numbers of MRV have been detected in peripheral B lymphocytes, which serve as a reservoir, maintaining a persistently covert infection (54). Similarly, it was reported that FV3, the type species of the genus *Ranavirus*, can be phagocytic and low copy harbored in macrophages/monocytes (55, 56). IHC studies have also suggested that the midwife toad virus (*Ranavirus alytes1*) infects liver cells, glomeruli, intestinal mucosa, and endothelial cells of young toads (9, 57). Despite these findings, global identification of ranavirus target cells remains underexplored. In this study, we revisited and characterized the histopathology of the pyloric ceca in detail. Using single-cell RNA sequencing (scRNA-seq), 18 distinct cell clusters were identified in the pyloric ceca, which were categorized into four groups: epithelial cells, stromal cells, immune cells, and red blood cells. After excluding the epithelial and muscle cells, the remaining cell population includes fibroblasts, myofibroblasts, and endothelial cells in the stromal group and B cells, T cells, macrophages, granulocytes, NK cells, and monocytes in the immune cell group. HIF plus FISH techniques and TEM observations identified fibroblasts, myofibroblasts, endothelial cells, B cells, and T cells as MRV-infected cells. TEM also revealed that MRV infected pericytes, although these cells were not identified in our scRNA-seq results due to the absence of specific pericyte markers. Based on the specific markers (PDGFRB) from other species, pericytes may be derived from smooth muscle cells or myofibroblasts. Given that myofibroblasts were identified as MRV target cells (Fig. S6), it is reasonable to infer that pericytes are also infected by MRV. Interestingly, MRV does not infect macrophages, which is consistent with our recent findings of persistent MRV infection in mandarin fish, but differs from persistent FV3 infection in *Xenopus laevis*, where peritoneal macrophages were confirmed as viral reservoirs (55, 56). In the mandarin fish model, peripheral B lymphocytes, but not T lymphocytes or macrophages, serve as the viral reservoirs during persistent MRV infection. Persistent MRV infection in fish is characterized by a long-term, low-copy, quiescent MRV state, which can be reactivated by environmental or pharmacological stressors (54). Notably, T lymphocytes are identified as MRV-targeted cells during acute infection but are non-permissive during persistent infection. We speculate that the robust T-cell-mediated immunity is sufficient to clear the low-copy quiescent MRV during persistent infection. Furthermore, our unpublished data indicate that the attenuated live MRV vaccine, but not the inactivated MRV vaccine, provides effective protection against virulent MRV challenge in the mandarin fish model. This suggests that cellular immunity, rather than humoral immunity, plays a crucial role in combating MRV infection. This may explain why MRV utilizes B cells, rather than T cells, as reservoirs to sustain persistent infections.

ScRNA-seq analysis of viral transcripts has been reported in several studies (58–60). However, when we applied scRNA-seq to analyze viral transcripts in our study, we encountered several limitations. Despite cross-referencing both the mandarin fish and MRV genomes, we found that only a small number of reads aligned with the viral genome, and these reads lacked cell specificity. Since scRNA-seq relies on capturing mRNA through magnetic beads that bind to the polyA tail, this method may miss MRV transcripts as they likely lack a polyA tail structure. Additionally, late-stage MRV infection leads to extensive cell lysis, which compromises the quality of the single-cell preparations, resulting in the loss of a significant number of viral target cells during sample processing. Furthermore, the accumulation of extracellular matrix components, particularly in the serosal layer, may hinder the accurate representation of viral infection sites in the cell populations obtained. These factors collectively limited our ability to analyze viral transcripts effectively, and as such, we did not include viral transcript data in our results. To address this issue, we employed alternative methods, such as dual-fluorescence immunofluorescence and electron microscopy, which allowed for a more direct and accurate visualization of virus-infected cells.

Virus infection and dissemination must overcome numerous obstacles, with the extracellular matrix (ECM) of host cells acting as a significant barrier. Some viruses have evolved strategies to bind to specific ECM components to facilitate infection (61). The ability of viruses to manipulate intercellular communication is crucial for successful infection as it results in the accumulation of viral particles on the host cell surface, promoting efficient binding to transmembrane or complex host cell receptors (62, 63). The ECM is a three-dimensional network of macromolecules including collagen, laminin, heparin sulfate, elastin, keratin, chondroitin sulfate, fibronectin, and hyaluronic acid which provide the environment and structure for biochemical support. Studies have shown that integrins, such as α1β1 and α2β1, act as receptors for rotavirus enterotoxins, while integrin α1β1 serves as a cell receptor for Ross River virus (64, 65). Furthermore, collagen-binding integrin VLA-1 modulates CD8^+^ T cell-mediated immune response against heterologous influenza virus infection (66). Collagen IV (col4a1 and col4a2), induced by human T-cell leukemia virus type 1 (HTLV-1) oncoprotein Tax, forms part of viral biofilms and influences viral transmission (67). In the present study, we observed that MRV infection of myofibroblasts and fibroblasts in the serosa of mandarin fish pyloric ceca promoted the expression of type V collagen. Additionally, MRV encoded four collagen-like proteins, which, together with host-encoded collagens, formed an ECM network that facilitated the recruitment of target and inflammatory cells. At later stages of infection, the disruption of the ECM and the infiltration of interstitial fluid aided in the rapid release of viral particles following viral replication.

**Fig 8:**
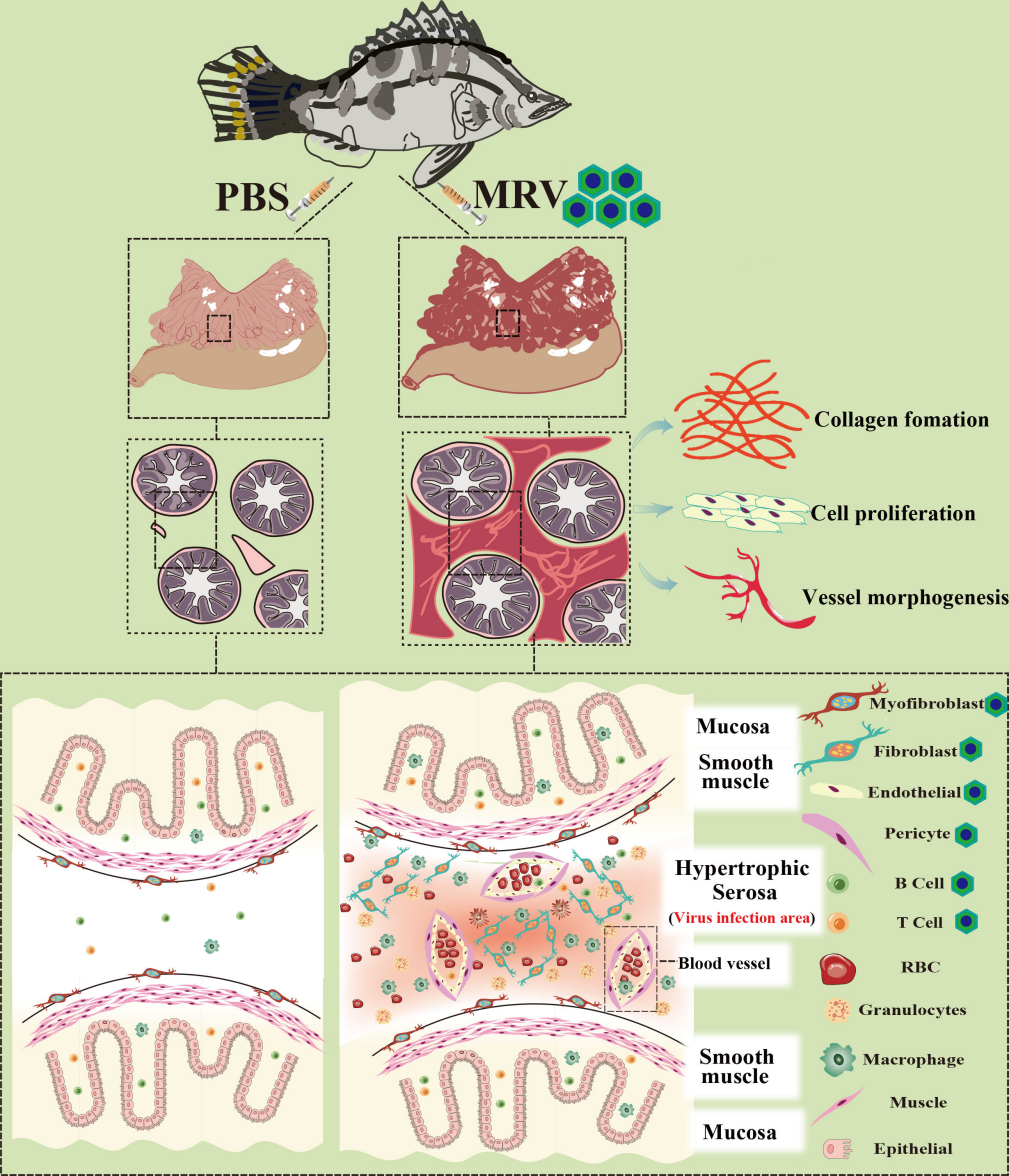
Diagram illustrating pyloric ceca lesions in mandarinfish upon acute MRV infection. The acute MRV infection results in severe fibrinous serositis characterized by severe congestion and edema in pyloric ceca, which is attributed to MRV primarily infecting mandarin fish serosa and resulting in pronounced serosal hyperplasia. Furthermore, MRV predominantly infected stromal components such as myofibroblasts, fibroblasts, endothelial cells, pericytes, and T and B lymphocytes within immune cells. This led to rapid ECM formation and recruitment of stromal and immune cells in the hypertrophic serosal zone, creating a favorable environment for viral replication. Simultaneously, due to the loose ECM structure, significant cell infiltration and interstitial fluid leakage resulted in severe ascites syndrome.

In summary, we established a comprehensive understanding of the pyloric cecum’s response to acute MRV infection in mandarin fish at single-cell resolution (Fig. 8). The key findings include the following: (a) the pyloric cecum serves as the tissue with the highest viral load, with the serosa layer bearing the primary infection; the acute MRV infection results in hyperplastic serosa and acute fibrinous serositis; (b) acute fibrinous serositis is characterized by the rapid aggregation of numerous inflammatory cells and the secretion of inflammatory factors; concurrently, a large number of stromal cells such as fibroblasts, myofibroblasts, endothelial cells, and pericytes were recruited to form the hypertrophic serosal zone; (c) T cells, B cells, fibroblasts, myofibroblasts, endothelial cells, and pericytes in the hypertrophic serosal zone are targeted by MRV, driving ECM formation, collagen encoding, and blood vessel morphogenesis; the infected pyloric ceca ultimately shows congestion, edema, and adhesions; (d) the loose ECM and the interstitial fluid infiltration facilitate the rapid release of the virus, further exacerbating the progression of serositis, leading to severe ascites and mortality. One limitation of this study is the relatively small number of biological replicates used for scRNA-seq, which constrained the depth of analysis and exploration of the single-cell data. This limitation stems from the lysis of cells during the terminal stages of viral infection (Fig. S7C), which results in the loss of some virus-infected cells during suspension preparation and may influence statistical analyses. Nevertheless, these limitations do not compromise the robustness of our conclusions regarding the identification of viral target cells or the mechanisms by which infection drives serositis.

## MATERIALS AND METHODS

### Virus, cells, and antibodies

MRV-ZQ17 strain was isolated and characterized from a natural mass mortality of juvenile mandarin fish and propagated in the mandarin fish fry (MFF-1) cell line (19). The MFF-1 cell line was grown in complete Dulbecco’s modified Eagle’s medium (DMEM) (Gibco, USA) containing 10% fetal bovine serum (FBS) (Gibco, USA), at 26°C under 5% CO_2_ incubation (68). Monoclonal antibodies (mAbs) specific to MRV 1C4 and mandarin fish IgM were constructed, characterized, and stored in our laboratory (20). The rabbit polyclonal antibodies of anti-MRV-011R, anti-MRV-033L, anti-MRV-067L, anti-MRV-068L, and anti-mandarin fish T-cell surface glycoprotein CD3delta were customized by GL Biochem (China). Additional rabbit polyclonal antibodies, including anti-actin alpha 2 (ACTA2) and anti-heat shock protein family b (small) member 1 (HSPB1), were obtained from ABMART company (China).

### Animals and artificial infection

Juvenile mandarin fish (average weight: ~45 g) were sourced from a fish farm in Foshan, Guangdong Province, China. The fish were confirmed to be free of MRV and ISKNV via qPCR, as previously described (20), and divided into two groups, with 30 fish per group. One group was used for MRV infection, while the other served as the mock-infected control. The water temperature was kept at 26 ± 0.5°C. For the infection group, an MRV-ZQ17 suspension cultured in MFF-1 cells was adjusted to a concentration of 10^7^ TCID_50_/0.1 mL and administered by intraperitoneal (i.p) injection at a dose of 0.1 mL per fish. The control group received an equivalent volume of sterile PBS. At 1 , 3, and 5 days post-infection (dpi), both infected and control fish were sampled for quantitative gene expression analysis, tissue tropism studies, and sc-RNA seq.

### Sample collection and dissection of scRNA-seq

Four pyloric cecum samples were collected, comprising two from MRV-infected fish and two from healthy controls. Single-cell suspensions were prepared from tissue fragments using a combination of enzymatic and mechanical dissociation. Cells capture and cDNA synthesis were carried out on a 10× Genomics platform. Transcriptome sequencing was performed on a NovaSeq 6000 platform after meeting all quality controls.

### Processing scRNA-seq data

Raw data generated with the 10× Genomics platform were aligned to the reference genome using Cell Ranger software (v7.0.1) to obtain the UMI matrix, which was further imported into R (v4.2.2) and processed with the Seurat package (v4.3.2). Cells with a detected gene number <200 or >5,000 or a high mitochondrial transcript ratio (>25%) were excluded. After normalization and scaling, the batch effect between infected and healthy fish was then removed using Harmony (69). Harmony is a computational algorithm designed to integrate multi-sample single-cell data sets by correcting batch effects across different experimental conditions. In this study, Harmony was employed to align gene expression data from infected and control groups, effectively minimizing technical variation and allowing for a more accurate comparison of biological differences between groups. The batch-corrected matrix was used for further analysis and visualization. The top 2,000 highly variable genes were extracted to perform principal component analysis (PCA), and the top 30 principal components (PCs) were used for cluster analysis. Cell type was annotated by the SingleR package (v1.2.4) and then checked manually.

### Identification of marker genes

To identify differentially expressed marker genes for each cell type, the Find All Markers function in Seurat was used under default parameters. Marker genes were selected as those with adjusted *P* values less than 0.05, average logFC larger than 0.25, and percentage of cells with expression higher than 0.25. Gene Ontology (GO), Kyoto Encyclopedia of Genes and Genomes (KEGG), and GSEA were performed with the R package, cluster Profiler v3.18.0, using all detected genes from the entire scRNA-seq library as background. Terms were enriched with the nominal *P* value < 0.05 and false discovery rate (q value) <0.05.

### Copy-number analysis

InferCNV (version 1.2.3; ref. 21) was used to infer large-scale copy number variations in immune cells using count data as input. Filtering, normalization, and centering by normal gene expression were performed using default parameters, and data were scaled. A cutoff of 0.1 was used for the minimum average read counts per gene among reference cells. An additional denoising filter was used with a threshold of 0.2. Copy-number variation was predicted using the default six-state hidden Markov model.

### Sample preparation, library construction, sequencing, and data analysis of ST

Fresh MRV-infected pyloric cecum was frozen using isopentane and liquid nitrogen bath. The frozen tissue samples were embedded with OCT and stored at −80°C. The frozen slides were carried out in the cryo-ultramicrotomes, and slides were collected for RNA extraction for quality control. RNA integrity number (RIN) values of ≥7 were required to confirm that RNA remained intact during tissue freezing. Tissue sections were mounted onto gene expression slides, followed by methanol fixation, hematoxylin and eosin (H&E) staining, and bright-field imaging. Tissue permeabilization was performed based on the permeabilization time determined during tissue optimization. Released mRNA from the tissue sections was captured by primers on the slides and reverse-transcribed into cDNA; the cDNA was collected from the slides and subjected to second-strand synthesis, denaturation, and PCR amplification. The amplified cDNA was fragmented enzymatically, end-repaired, A-tailed, and purified using beads. Fragments were screened, adapters ligated, and purified again before indexing through PCR to construct a standard next-generation sequencing (NGS) library. Qualified libraries were sequenced using an NGS platform in the paired-end 150 (PE150) mode. Sequencing data were processed using BSTMatrix software (Biomarker tech., China) for BMKMANU S1000 spatial transcriptome sequencing. The likely single-cell composition of each spot was determined by carrying out factor analysis using the scRNA-seq atlas as a reference, thus spatially localizing all scRNA-seq clusters.

### Viral load measurement by absolute qPCR

The MRV genome copy number was determined by absolute qPCR. DNA templates were extracted from different mandarinfish tissues at 5 dpi using the FastPure Cell/Tissue DNA Isolation Mini Kit (Vazyme, China) according to the manufacturer’s instructions. An absolute standard curve of the MRV *mcp*-specific qPCR system was established through diluted pMD-19T-MCP recombinant plasmid DNA. The reactions were performed using the Light Cycler 480-II Multiwell Plate 384 real-time detection system (Roche Diagnosis, the USA) under the following conditions: 1 cycle at 95°C for 60 s, and 40 cycles at 95°C for 5 s, 60°C for 30 s, and 70°C for 5 s with a total reaction volume of 10 µL, containing 1 µL of DNA, 5 µL of Real Time PCR Easy-Taqman (Foregene, China), 0.5 µM primers, 0.2 µM probe (Tsingke, China), and 3 µL of RNase free water. All real-time qPCRs were performed in triplicate.

### Quantitative gene expression analyses

Total RNA from various tissues of infected mandarin fish (*n* = 3) at 0, 1, 3, and 5 dpi were extracted using the Eastep Super Total RNA Extraction Kit (Promega, China) according to the manufacturer’s instruction. RNA was reverse-transcribed into cDNA using Evo M-MLV RT Premix for qPCR (Accurate Biology, China). The gene expression levels were quantified by qRT-PCR methods in the Roche LightCycler 480 system, with mandarin fish β-actin as the reference gene. Gene-specific primers were designed and validated by gradient PCR and with the qPCR melting curves. All primers used in this study are listed in Table S2. The qRT-PCR was performed as described previously (19). Briefly, PCRs were performed as the following procedure: 95°C for 5 min, one cycle; 95°C for 5 s, 60°C for 30 s and 70°C for 5 s, 40 cycles; 95°C for 5 s, 60°C for 1 min and 95°C, one cycle; 50°C for 30 s, one cycle with a total reaction volume of 10 µL, containing 1 µL of cDNA, 5 µL of 2 × SYBR Green Pro Taq HS Premix (Accurate Biology, China), 0.5 µM primers (Tsingke, China), and 3.6 µL of RNase-free water.

### Histopathology, immunofluorescence assay (IFA), and immunohistochemistry (IHC)

Tissues from three moribund fish were dissected and fixed with alcohol-formalin-acetic acid (AFA) for hematoxylin-eosin (H&E) staining or in 4% paraformaldehyde for IFA and IHC analysis. The fixed tissues were embedded and excised into sections, dewaxed in xylene, and rehydrated in a series of ethanol solutions, according to protocols described in our previous reports (19). For IHC analysis, the sections were performed using mouse anti-MRV mAb of 1C4 (1:1,000) as the primary antibody and horseradish peroxidase (HRP)-labeled goat anti-mouse IgG as the second antibody and then developed with 3,3 N-diaminobenzidine tetrahydrochloride (DAB) solution. Sections were visualized under a Nikon fluorescence microscope (Eclipse Ni-E, Japan). The IHC image in Fig. 3C was partially derived from a magnified section of the whole-slide scan shown in Fig. 1F, allowing a detailed observation of the viral distribution in specific anatomical regions such as the digestive tract. For simple or double staining IFA (Fig. 1, 5, 6B, 7A and E), the sections were stained with primary anti-MRV monoclonal antibody and corresponding polyclonal antibody at 1:1,000 dilutions and secondary Alexa Fluor 555-labeled monkey anti-mouse IgG antibody and Alexa Fluor 488-labeled goat anti-rabbit IgG antibody at 1:1,000 dilution and then stained by 4′,6- diamidino-2-phenylindole (DAPI) (Abcam, China). For dual-anti-mouse double staining IFA (Fig. 6B-IgM), the sections were stained with the Three-color Multiplex Immunohistochemical Kit (Shanghai YEPCOME Biotechnology Co., Ltd, China) according to the manufacturer’s instruction. Finally, sections were visualized under a confocal laser scanning immunofluorescence microscopy (Leica SP8, German).

### Fluorescent *in situ* hybridization (FISH) and IFA

FISH sweAMI riboprobes and corresponding branch probe targeting the open reading frame (ORF) sequences of *CDH5*, *ALOXE3*, *CSF1RB*, *HBβ,* and *EPCAM* genes were customized from Servicebio (Servicebiotech, China). The riboprobe sequences are provided in Supplementary S1 (Table S1). Pyloric ceca were rinsed with PBS and immediately put into *in situ* hybridization fixative (Servicebiotech, China) for over 12 h and stored at 4°C. After fixation, the target tissue blocks were sectioned to approximately 3 mm thickness under a fume hood, dehydrated through a graded ethanol series, and cleared with xylene. The tissue blocks were embedded in paraffin and sectioned into 4 µm slices, which were baked at 62°C for 2 hours. After dewaxing, dehydration and antigen retrieval, and pre-hybridization at 37°C for 1 h, the sections were hybridized overnight in a constant temperature chamber. After hybridization, sections were sequentially washed twice in 2 × saline-sodium citrate (SSC) (1 × SSC = 0.15 M NaCl, 15 mM Na citrate) at room temperature for 15 min and then in 1 × SSC and 0.1 × SSC at 55°C for 1 h. Branch probes were applied for hybridization at 40°C for 45 minutes. After washing, CY5-labeled signal probes were added and incubated at 42°C for 3 hours. Following FISH, tissue sections were stained with primary anti-MRV monoclonal antibody, followed by secondary Alexa Fluor 488-labeled monkey anti-mouse IgG antibody at 1:1,000 dilution and then stained by 4′,6- diamidino-2-phenylindole (DAPI) (Abcam, China). Sections were visualized under a Nikon fluorescence microscope (Eclipse Ni-E, Japan).

### Transmission electron microscopy (TEM)

TEM analysis was performed as per our previous description (18). MRV-infected pyloric ceca from moribund mandarin fish at 5 dpi were collected for TEM assays. Briefly, infected tissues were fixed with 2.5% glutaraldehyde in 0.1 M PBS, followed by secondary fixation in 2.0% osmium tetroxide in 0.1 M PBS. After dehydrating, penetrating, embedding, and polymerization, ultrathin sections (60 nm) were prepared. Sections were stained with uranyl acetate-lead citrate and examined under a Philips CM10 electron microscope.

### Statistical analysis

Analysis of variance (ANOVA) was performed for statistical analysis of expression and viral load data. Statistical analysis of survival data were performed using a Log-Rank Test (GraphPad Prism 8, San Diego, CA, USA). * means *P* < 0.05; ** means *P* < 0.01, *** means *P* < 0.001, **** means *P* < 0.0001, ns means no significance. Error bars on all graphs represent the standard error of the mean (SEM).

## Data Availability

The raw ScRNA-seq data have been deposited in the NCBI database under accession number PRJNA1225909. The authors confirm that the data supporting the findings of this study are available within the article and/or its supplemental material.

## SUPPLEMENTAL MATERIAL

Figure S1 jvi.02308-24-s0001.tif Temporal changes of infected pyloric ceca during acute MRV infection.

Figure S2 jvi.02308-24-s0002.tif Data quality control of scRNA-seq.

Figure S3 jvi.02308-24-s0003.tif UMAP and violin plots illustrating the expression of representative marker genes.

Figure S4 jvi.02308-24-s0004.tif UMAP plots illustrating marker gene expression in epithelial and stromal cell clusters.

Figure S5 jvi.02308-24-s0005.tif Expression of representative marker genes and the DEGs of immune cells.

Figure S6 jvi.02308-24-s0006.tif Spatial transcriptomics analysis of MRV-infected pyloric caeca.

Figure S7 jvi.02308-24-s0007.tif Comparison of GO terms for MRV target cells between control and MRV-infected mandarin fish.

Supplemental legends jvi.02308-24-s0008.docx Legends for supplemental material.

Table S1 jvi.02308-24-s0009.xlsx Underlying numerical data for Fig. 4H.

Table S2 jvi.02308-24-s0010.xlsx Primer and probe sequences.

ASM does not own the copyrights to Supplemental Material that may be linked to, or accessed through, an article. The authors have granted ASM a non-exclusive, world-wide license to publish the Supplemental Material files. Please contact the corresponding author directly for reuse.
