# Supplementary figures and images for: Dissection of the global responses of mandarin fish pyloric cecum to an acute ranavirus (MRV) infection reveals the formation of serositis and then ascites
Source: J Virol. 2025 May 14;99(6):e02308-24. doi: 10.1128/jvi.02308-24 (PMC12172472; doi:10.1128/jvi.02308-24)

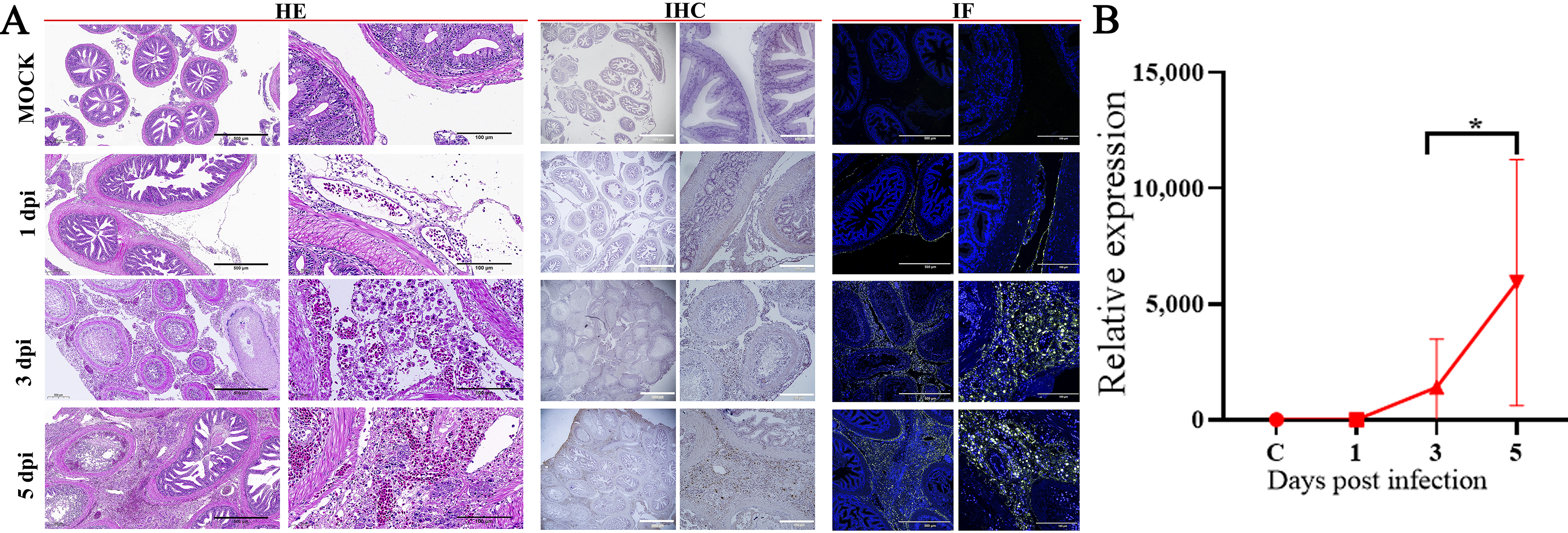

Supplement: Figure S1 — Temporal changes of infected pyloric ceca during acute MRV infection. [file jvi.02308-24-s0001.tif]

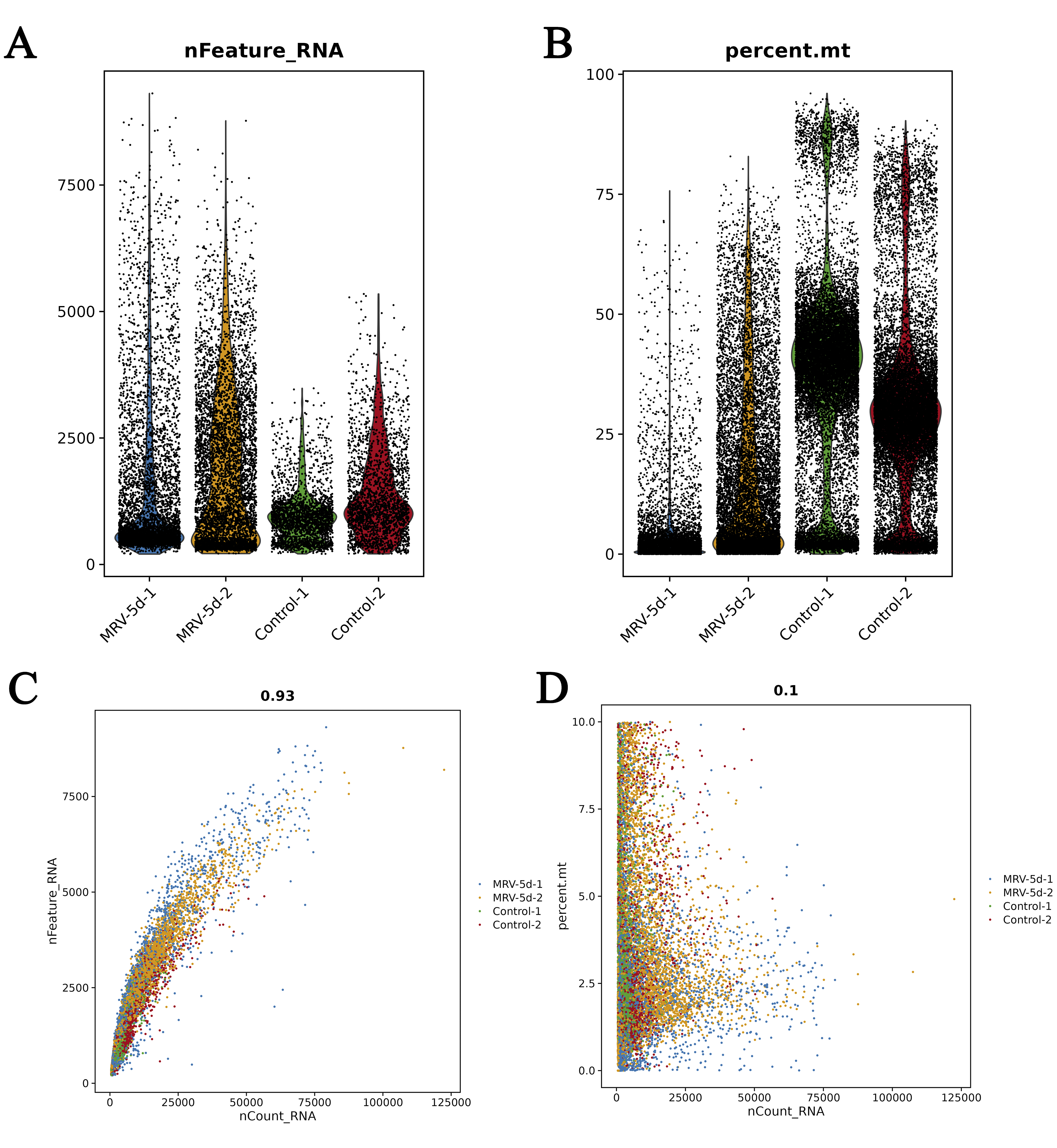

Supplement: Figure S2 — Data quality control of scRNA-seq. [file jvi.02308-24-s0002.tif]

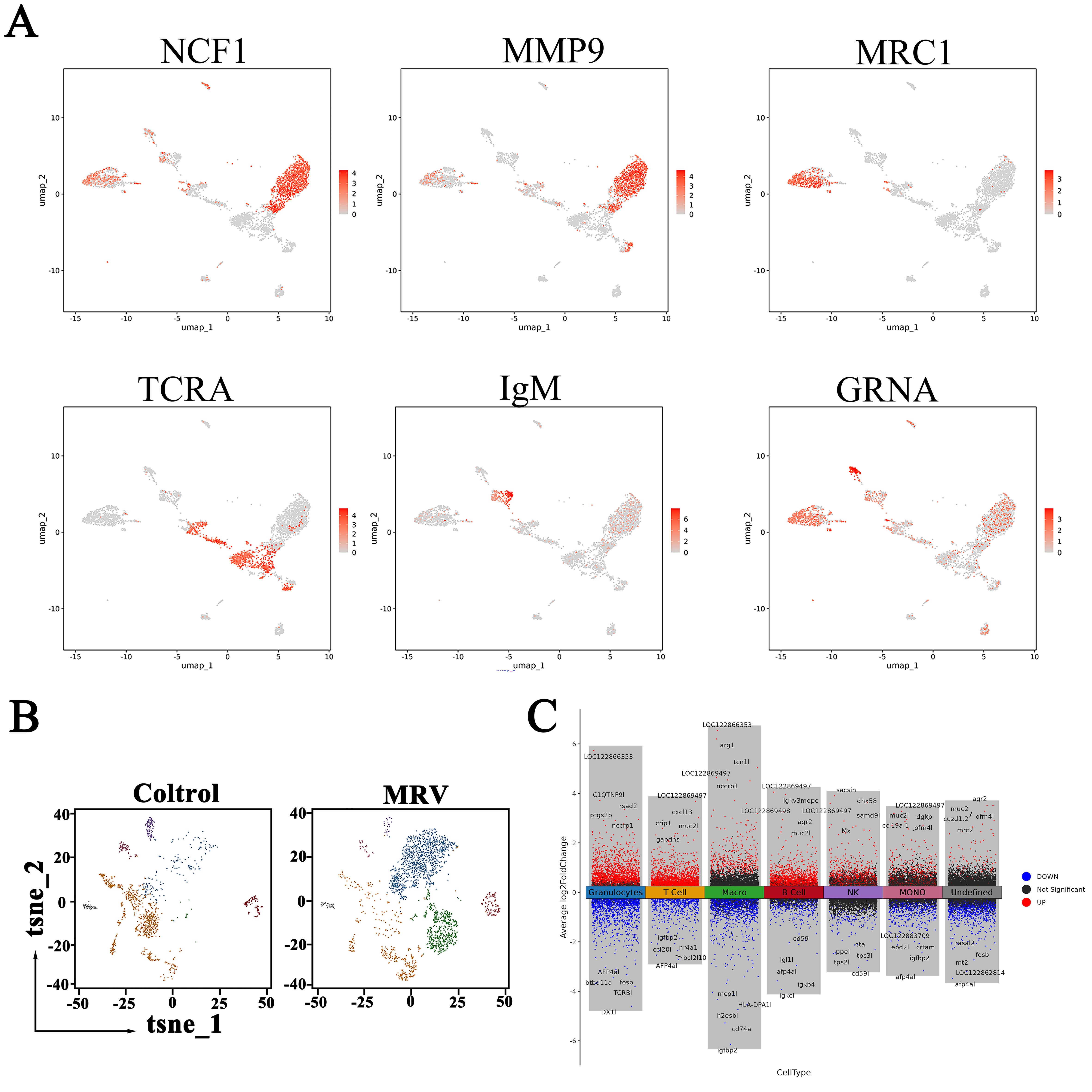

Supplement: Figure S5 — Expression of representative marker genes and the DEGs of immune cells. [file jvi.02308-24-s0005.tif]

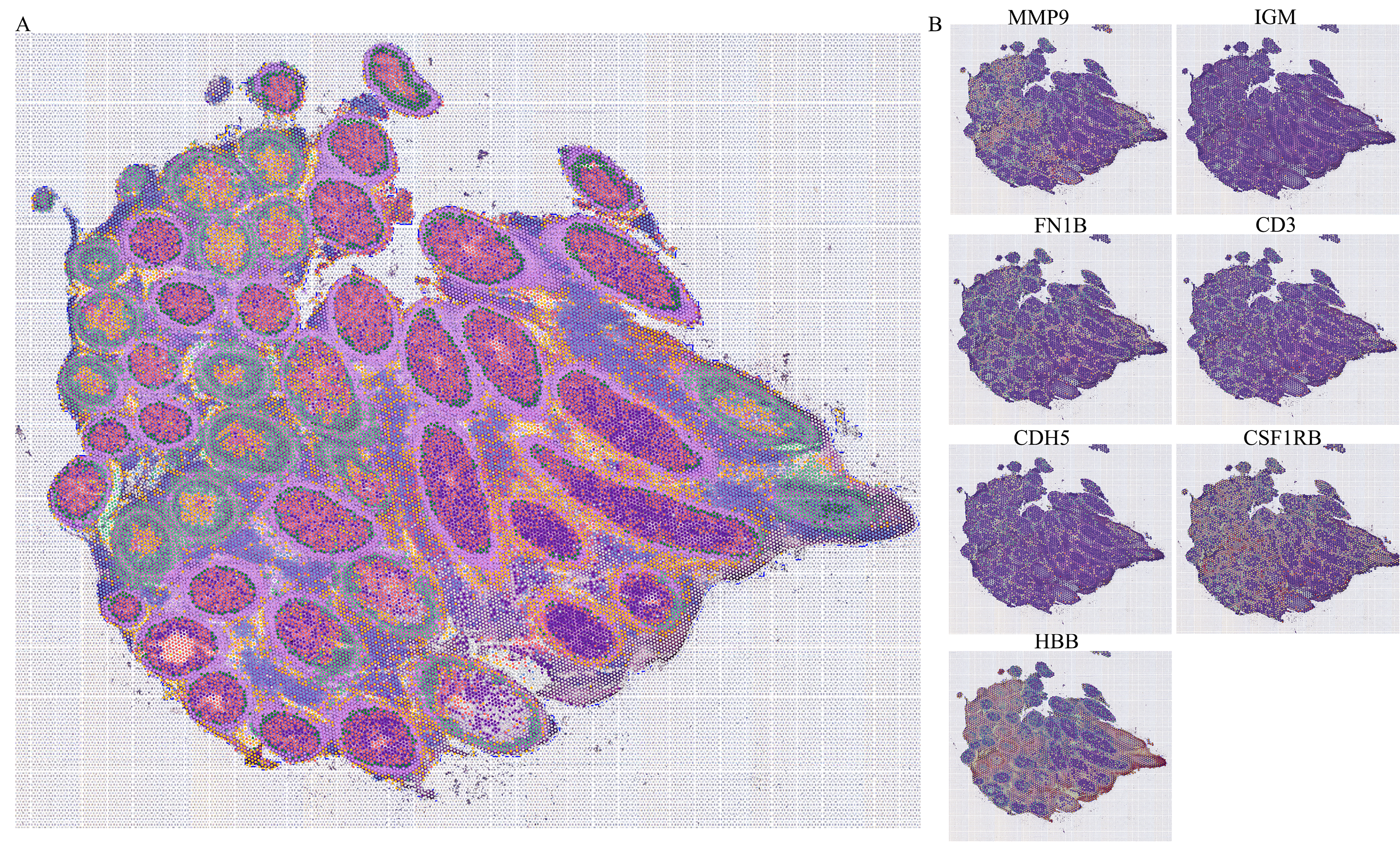

Supplement: Figure S6 — Spatial transcriptomics analysis of MRV-infected pyloric caeca. [file jvi.02308-24-s0006.tif]

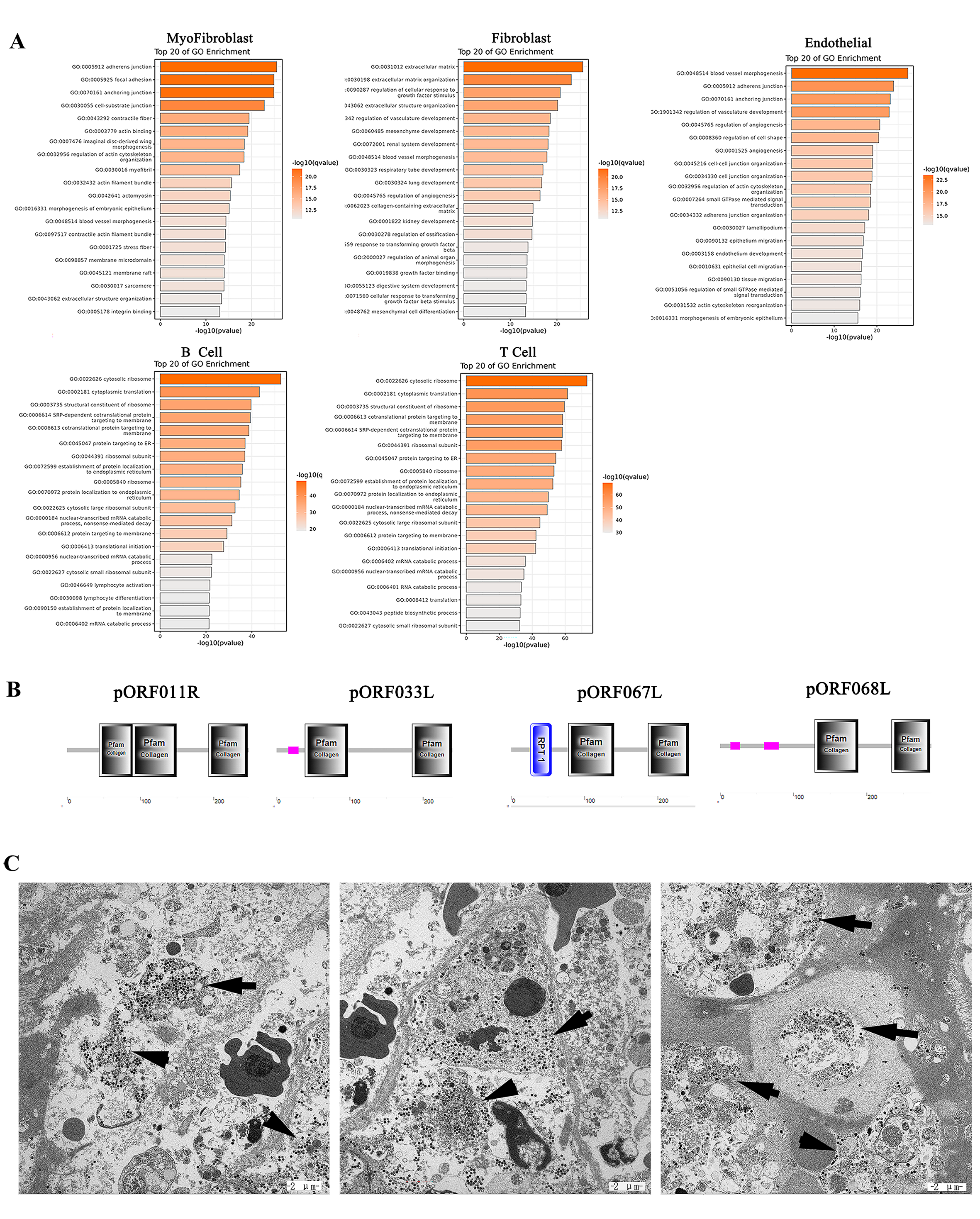

Supplement: Figure S7 — Comparison of GO terms for MRV target cells between control and MRV-infected mandarin fish. [file jvi.02308-24-s0007.tif]
