# Supplementary material for: Dissection of the global responses of mandarin fish pyloric cecum to an acute ranavirus (MRV) infection reveals the formation of serositis and then ascites
Source: J Virol. 2025 May 14;99(6):e02308-24. doi: 10.1128/jvi.02308-24 (PMC12172472; doi:10.1128/jvi.02308-24)
Supplement: Supplemental legends — Legends for supplemental material. [file jvi.02308-24-s0008.docx]

**Supporting Information**

**Fig S1. Temporal changes of infected pyloric caeca during acute MRV infection.** (A) (Left), Histopathological changes of infected pyloric caeca visualized by H&E staining at 1, 3 and 5 dpi, showing progressive serositis classified as slight, mild, and severe. The interface between healthy pyloric caecum is clear, with minimal mesenchymal infiltration. (Middle), IHC analysis illustrating MRV distribution in the pyloric caeca over the infection timeline. (Right), IF imaging tracing the progression of MRV infection in the pyloric caeca. Scale bars are shown in the figure. (B) The relative expression level of the *mcp* gene of MRV.

**Fig. S****2 Data quality control of scRNA-seq.** (A) Distribution of gene number detected in single cell across 4 samples (Y axis). (B) Distribution of the percentage of mitochondrial gene expression in single cell across 4 samples (Y axis). (C) Relationship between the number of nUMI and nGene. (D) Relationship between the number of nUMI and pMito.

**Fig. S3 UMAP and violin plots illustrating the expression of representative marker genes.** UMAP and violin plots show that the expression of representative marker genes is restricted to specific clusters among all cells.

**Fig. S4. UMAP plots illustrating marker gene expression in epithelial and stromal cell clusters.**

(A) UMAP plots showing that the expression of representative marker genes is restricted to specific clusters within epithelial subtype cells.

(B) UMAP plots showing that the expression of representative marker genes is restricted to specific clusters within stromal subtype cells.

**Fig. S5 the expression of representative marker genes and the DEGs of immune cells.** (A) Umap plots showing that representative marker genes are restricted to specific immune cell clusters. (B) tSNE plots aligning clusters of immune subtype cells between control and MRV-infected mandarin fish. (C) Volcano plot illustrating DEGs in immune cells between control and MRV-infected groups. Genes specifically upregulated or downregulated are highlighted in red and blue, respectively. Each dot represents an individual gene, with significant genes identified by adjusted p < 0.05 (adjusted by false discovery rate in MAST). Non-significant genes are shown in gray.

**Fig. S6. Spatial transcriptomics analysis of MRV-infected pyloric caeca.**

(A) Spatial transcriptomics data showing the distribution of cell clusters in pathological sections of MRV-infected pyloric caeca.

(B) Expression of selected cell markers identified by scRNA-seq in MRV-infected pyloric caeca.

**Fig. S7** (A) Comparison of GO terms for MRV target cells between control and MRV-infected mandarin fish, labeled with names and IDs and sorted by −log10 (P) value. The top 20 enriched GO terms are shown. (B)Prediction of four MRV-encoded collagen-like protein domains using SMART. (C) TEM images showing the lytic cells at the end stage of infection. Arrows indicated the MRV virions.

**Table S1** The underlying numerical data for Fig 4H.

**Table S2** Primers used in the RT-qPCR and the sequence used for synthesis SweAMI FISH riboprobes.
